# Genomic signatures of environmental selection despite near‐panmixia in summer flounder

**DOI:** 10.1111/eva.12676

**Published:** 2018-08-17

**Authors:** Jennifer A. Hoey, Malin L. Pinsky

**Affiliations:** ^1^ Department of Ecology, Evolution, & Natural Resources Rutgers University New Brunswick New Jersey USA

**Keywords:** adaptation, ddRADseq, genome scan, population structure, spatial balancing selection, summer flounder

## Abstract

Rapid environmental change is altering the selective pressures experienced by marine species. While adaptation to local environmental conditions depends on a balance between dispersal and natural selection across the seascape, the spatial scale of adaptation and the relative importance of mechanisms maintaining adaptation in the ocean are not well understood. Here, using population assignment tests, Approximate Bayesian Computation (ABC), and genome scans with double‐digest restriction‐site associated DNA sequencing data, we evaluated population structure and locus–environment associations in a commercially important species, summer flounder (*Paralichthys dentatus*), along the U.S. east coast. Based on 1,137 single nucleotide polymorphisms across 232 individuals spanning nearly 1,900 km, we found no indication of population structure across Cape Hatteras, North Carolina (*F*_ST_ = 0.0014) or of isolation by distance along the coast using individual relatedness. ABC estimated the probability of dispersal across the biogeographic break at Cape Hatteras to be high (95% credible interval: 7%–50% migration). However, we found 15 loci whose allele frequencies were associated with at least one of four environmental variables. Of those, 11 were correlated with bottom temperature. For summer flounder, our results suggest continued fisheries management as a single population and identify likely response mechanisms to climate change. Broadly speaking, our findings suggest that spatial balancing selection can manifest in adaptive divergence on regional scales in marine fish despite high dispersal, and that these conditions likely result in the widespread distribution of adaptive alleles and a high potential for future genetic adaptation in response to changing environmental conditions. In the context of a rapidly changing world, a landscape genomics perspective offers a useful approach for understanding the causes and consequences of genetic differentiation.

## 1 INTRODUCTION

The rapid rate of environmental change is altering the selective pressures experienced by species and threatening global biodiversity (McCauley et al., 2015; Sala et al., 2000). Understanding species’ responses to environmental change and determining which species will persist into the future, and which will not, are major challenges in the fields of ecology and evolution. In the ocean, populations of species can persist by moving in response to shifting conditions, such as climate (Pinsky, Worm, Fogarty, Sarmiento, & Levin, 2013; Poloczanska et al., 2013), or by adapting in situ through genetic changes or phenotypic plasticity (Crozier & Hutchings, 2014; Hoffmann & Sgro, 2011; Hollander, 2008). Distinguishing between environmental‐induced genetic adaptation and phenotypic plasticity is challenging (Merilä & Hendry, 2014; Reusch, 2014), but populations can respond quickly to environmental cues through both mechanisms (Hendry, Farrugia, & Kinnison, 2008; Kovach, Gharrett, & Tallmon, 2012; Miller et al., 2012; Reznick, Bryga, & Endler, 1990; Reznick & Ghalambor, 2001). The means by which species respond to changing environments are determined by the potential for dispersal, the strength of selection, and the scale of environmental heterogeneity (Levins, 1968). Thus, understanding marine species’ responses to environmental change requires knowledge of how phenotypic and genotypic spatial variations are shaped by the dispersal‐selection balance across the seascape.

Our understanding of the spatial scale of genetic mixing in marine species has been undergoing a paradigm shift. The majority of marine species have historically been viewed as highly connected, well‐mixed populations by virtue of their high dispersal rates, lengthy pelagic larval durations, and the assumption that larvae act as relatively passive particles (Siegel, Kinlan, Gaylord, & Gaines, 2003). Despite the positive correlation between pelagic duration and dispersal distance (Shanks, 2009), evidence has emerged that many wide‐ranging marine species exhibit fine‐scale population substructure at neutral loci (Benestan et al., 2015; Therkildsen et al., 2013) or are locally adapted to their surrounding conditions at loci experiencing natural selection (Conover, Clarke, Munch, & Wagner, 2006; Pespeni & Palumbi, 2013; Sanford & Kelly, 2011; Therkildsen et al., 2013). These lines of evidence suggest that marine populations are not as homogenous as previously thought (Hedgecock, 1986). For instance, species occupying a wide geographic area characterized by an environmental cline can become genetically matched to their local environment if dispersal is low enough or, alternatively, if selection is strong enough. Thus, spatial local adaptation (Kawecki & Ebert, 2004) or spatial balancing selection (Hedrick, Ginevan, & Ewing, 1976; Sotka, 2012; Whitlock, 2015), which is sometimes called spatially varying selection (Bernatchez, 2016), can result in genetic divergence at particular loci between populations occupying different habitats. This is important because, populations harboring adaptive polymorphisms across a heterogeneous landscape can serve as repositories of genetic variation, contributing potentially beneficial alleles to other populations along the cline, and enabling species persistence as environmental conditions shift.

Summer flounder (*Paralichthys dentatus*) support an economically important commercial and recreational fishery and inhabit estuarine and continental shelf waters characterized by environmental differences from Nova Scotia, Canada to Florida, USA. In particular, the warm Gulf Stream flows northward from Florida, hugging the coast until it spirals offshore at Cape Hatteras, North Carolina, creating a steep thermal gradient. Summer flounder exhibit a seasonal inshore–offshore migration pattern. Adults and juveniles reside in shallow coastal and estuarine waters during the summer months before moving offshore in the fall and early winter, when spawning occurs. Both adult and juvenile fish remain on the continental shelf throughout the winter and then return to coastal and estuarine waters in the late spring to early summer (O'Brien, Burnett, & Mayo, 1993; Packer et al., 1999). Overfishing of summer flounder resulted in a sharp population decline in the late 1980s and early 1990s, prompting severe fishing restrictions and conservation measures to improve stock abundance (Terceiro, 2011). Today, the summer flounder stock has been successfully rebuilt and is managed as a single population with state allocations of fisheries quota based on the 1980s population distribution. The center of summer flounder abundance lies offshore of the Mid‐Atlantic states, between Cape Cod, Massachusetts and Cape Hatteras, North Carolina (Packer et al., 1999). Recent studies have suggested that summer flounder biomass has shifted within the Mid‐Atlantic, due either to changing climate (Nye, Link, Hare, & Overholtz, 2009; Pinsky & Fogarty, 2012) or as a response to fishing pressure (Bell, Richardson, Hare, Lynch, & Fratantoni, 2014). Regardless of mechanism, shifts in summer flounder biomass have management consequences because of a growing mismatch between state allocations in quota and the geographic distribution of summer flounder biomass. Even though some fisheries can track shifting species, the disparity between the locations of fish and fishermen is likely to have socioeconomic consequences (Pinsky & Fogarty, 2012). The situation may be further complicated if population genetic structure exists, as state allocations would be affecting different spawning stocks.

Past studies on population structure in summer flounder have found differences in allozymes, morphology, meristic traits, and timing of shallow water ingress between individuals caught north and south of Cape Hatteras, suggesting the existence of multiple spawning stocks, or subpopulations (Able, Matheson, Morse, Fahay, & Shepherd, 1990; Able et al., 2011; Burke, Monaghan, & Yokoyama, 2000; Kraus & Musick, 2000; Van Housen, 1984). However, another study found no genetic differentiation between supposed subpopulations after examining mitochondrial DNA (Jones & Quattro, 1999), so summer flounder population substructure remains equivocal. With the increasing ease of sampling hundreds to thousands of loci across the genome, reduced representation sequencing in summer flounder can provide a more robust estimate of population structure, as well as assess genetic variation across spatially divergent environmental conditions.

In this study, we use double‐digest restriction‐site‐associated DNA (ddRAD) sequencing to generate 1,137 single nucleotide polymorphisms (SNPs) genotyped in 232 adult summer flounder individuals captured along the U.S. east coast. Using these data, we address two questions: (i) Do summer flounder exhibit population structure and (ii) are there particular loci that suggest locally divergent environmental selection? Our results contribute to a growing number of studies suggesting that a balance between multiple evolutionary forces shapes the genetic makeup of marine populations and that understanding these forces is important for the persistence and management of species under future climate change.

## 2 METHODS

### 2.1 Sample collection

**Figure 1:**
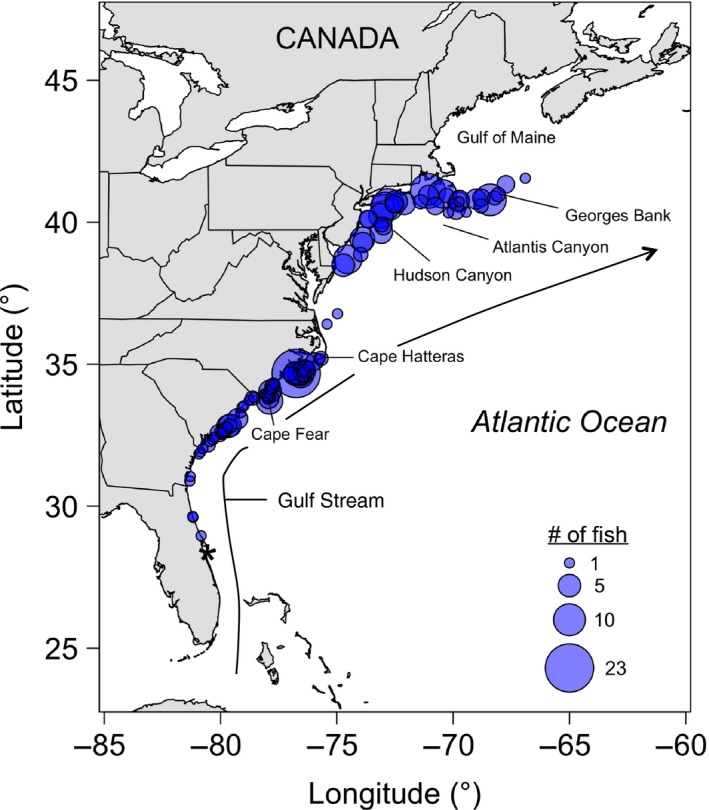
Sampling locations of adult summer flounder (*n *=* *306) from bottom trawl surveys and recreational sampling along the U.S. east coast. Circle area reflects sampling size at each location. Important geographic and oceanographic features are also noted. The * indicates the location (28.3849, ‐80.5463) from which “distance along the coast” was calculated for locus–environment associations

Regional bottom trawl surveys in 2013–2014 collected adult summer flounder from Massachusetts to Florida (Table 1 and Figure 1): the Southeast Area Monitoring and Assessment Program (SEAMAP; *n *=* *123) during Fall 2013 – Spring 2014 and the Northeast Fisheries Science Center Fall Bottom Trawl Survey (NEFSC FBTS; *n *=* *153) during Fall 2014. These were augmented by specimens collected by the North Carolina Department of Natural Resources (NCDNR; *n *=* *30) from recreational anglers fishing on artificial reefs during summer 2014. Total length of specimens ranged from 17.4 to 76.5 cm, with an average of 39.3 cm and a median of 38.6 cm. All individuals from the NEFSC FBTS were classified as mature, but information on maturity was not available for the SEAMAP nor NCDNR individuals. Previous published estimates of median length of maturity for summer flounder range from 28 to 32 cm for females and 24.9 to 28.9 cm for males (Morse, 1981; O'Brien et al., 1993; Wenner et al., 1990). Based on these estimates, some SEAMAP and NCDNR individuals may have been immature, but mature and immature summer flounder move offshore and overwinter together (Packer et al., 1999). For all samples (*n *=* *306), a small muscle tissue plug and/or fin clip was removed and preserved in 95% ethanol. Tissue and fin clips were taken upon capture for NEFSC FBTS and NCDNR specimens. SEAMAP specimens were shipped frozen to Rutgers University, where tissue and fin clip samples were taken after thawing. All tissue and fin clip samples were held at ‐20°C until DNA extraction.

**Table 1:** Sampling year, season, source, latitudinal range, sample size, and range of total lengths (TL) for each collection of adult summer flounder. The range of total lengths reflects available data for each collection

Year	Season	Source	Latitudinal range (°)	*N*	TL Range (cm)
2013	Fall	Southeast Area Monitoring & Assessment Program (SEAMAP)	28.96–35.20	54	25.3–40.0
2014	Spring	Southeast Area Monitoring & Assessment Program (SEAMAP)	31.83–35.19	69	17.4–43.1
2014	Summer	North Carolina Department of Natural Resources (NCDNR)	34.56–34.70	30	38.7–48.3
2014	Fall	Northeast Fisheries Science Center (NEFSC) Fall Bottom Trawl Survey	36.41–41.55	153	35.5–76.5

### 2.2 DNA extraction

To extract whole genomic DNA from adult summer flounder, ≤ 20 mg of muscle tissue or fin was lysed, and DNA was washed and eluted using DNeasy 96 Blood & Tissue Kits (QIAGEN; Hilden, Germany) and the manufacturer's recommended protocols. DNA extracts were visualized on 2% agarose gels to assess quality and were subsequently quantified using PicoGreen (Thermo Fisher Scientific, Waltham, MA, USA) and a SpectraMax M3 Microplate Reader (Molecular Devices; Sunnyvale, CA, USA).

### 2.3 Library preparation and sequencing

Summer flounder ddRAD libraries were prepared according to a protocol adapted from Peterson, Weber, Kay, Fisher, and Hoekstra (2012). In brief, successful extracts were digested in 50 μl reactions using PstI and EcoRI restriction enzymes for 4 hr at 37°C. Digested samples were cleaned with AMPure beads (Beckman Coulter; Brea, CA, USA) to remove small DNA fragments less than 100 base pairs (bp) in size and any remaining proteins, including restriction enzymes. Cleaned digestions were then quantified again with PicoGreen and a SpectraMax M3 Microplate Reader. High‐quality digested samples were sorted by DNA quantity and grouped into pools of up to 48 individual samples for ligation of P1 adapters containing unique 6‐nucleotide barcodes and a P2 forked adapter. P1 adapters were designed to adhere to the sticky end left by PstI while the P2 adapter was designed to ligate to the overhang left by EcoRI. Each sample grouped into a pool was individually ligated using X ng of digested DNA in 30 μl reactions, where X was 10‐200, depending on the pool. Ligation reactions occurred at 25°C for 1.5 hr, after which the enzyme was heat‐killed at 65°C for 10 min, followed by a decrease of 2°C every 90 s until room temperature was achieved. Ligated samples were then pooled and cleaned twice with AMPure beads. Pooled samples were size selected to 273 ± 27 bp using a Pippin Prep (Sage Science; Beverly, MA) and then amplified with PCR using read 1 and read 2 primers (Table S1) designed to only amplify DNA with both P1 and P2 adapters. Read 2 PCR primers contained one of 12 Illumina indices so that pools could be distinguished from one another. AMPure beads were used to clean away extraneous proteins and small‐sized DNA. Qubit Fluorometric Quantitation (Thermo Fisher Scientific; Waltham, MA, USA) was used to quantify the final concentration of each pool. An initial 10 nM library composed of two pools containing 20 individuals each was sent to the Rutgers Genome Cooperative (New Brunswick, NJ, USA) for 200 bp single‐end sequencing on the Illumina Miseq platform. A subsequent 10 nM library of five pools containing 48 individuals each was sent to the Princeton Core Facility (Princeton, NJ, USA) for 141 bp single‐end sequencing on a single lane run using the Illumina HiSeq 2500 platform. In total, we sequenced of 280 adult summer flounder.

### 2.4 Bioinformatics and genotyping

To distinguish between pooled libraries, sequenced reads were demultiplexed by Illumina index using a Python script adapted from FASTX Barcode Splitter in the FASTX toolkit programs (Gordon, 2011). To determine reads from particular individuals, sequenced reads were further demultiplexed by barcode and cleaned using process_radtags in STACKS v.1.29 (Catchen, Hohenlohe, Bassham, Amores, & Cresko, 2013). Next, sequences were run through the dDocent v. 2.14 pipeline (Puritz, Hollenbeck, & Gold, 2014), an analysis software for ddRAD sequencing data that trims, assembles, maps, and calls SNPs. In brief, using default dDocent settings unless otherwise noted, reads were trimmed for quality using TrimGalore! *De novo* single‐end assembly of reference sequences was performed with Rainbow using alleles with a minimum within‐individual coverage level of 2.5 and a minimum occurrence in 15 individuals. Reference sequences with ≥90% similarity were clustered together with CD‐HIT. Individual quality‐trimmed reads were then mapped to the reference sequences using BWA, and SNPs were identified with FreeBayes.

Variant SNPs that were successfully genotyped in at least 50% of individuals with a minor allele count of at least three were retained in the analysis. To remove individuals that did not sequence well, any genotypes with fewer than three reads were recoded as “missing.” About 15% of individuals with the most missing data, corresponding to individuals with more than 77% missing data, were discarded. This filter left 241 of 280 individuals for further analysis. Data were then restricted to variants occurring in 95% of individuals with a minor allele frequency (MAF) of 0.05 and a minimum mean depth of 20. Further filtering was conducted using a filtering script distributed with dDocent that filters reads based on allele balance, locus quality, mapping quality, depth, and strand representation. First, variable sites were removed if the average allele balance at heterozygous genotypes was less than 25%, the ratio of quality score to depth was less than 0.2, and the ratio between the mean mapping quality of the alternate and reference alleles was less than 0.25 or more than 1.75. The average depth and standard deviation were then calculated across all individuals for all remaining sites. Sites with depths greater than the average depth plus one standard deviation were removed if the quality score was less than double the depth. At last, to filter out sites with unreasonably high mean depth, we calculated mean depth and kept the lower 95% of the distribution, corresponding to a mean depth of 88 or less. Indels were then removed, and only the first SNP at each contig was retained to help ensure an unlinked dataset. Hence, we will hereafter refer to these SNPs as loci.

From initial investigations of the dataset, we discovered 10 very closely related summer flounder based on Rousset's distance that were caught in the same tow. No other tow exhibited a similar degree of genetic similarity. These fish were not siblings based on analysis in COLONY v.2.0.6.3 (Jones & Wang, 2010), but could be the result of cross‐contamination in the trawl net. We excluded 9 of 10 of these individuals for the analyses presented in the paper, but also tested whether our findings were sensitive to this choice by performing the analyses with all individuals included. Even with all individuals included, we found no genomewide population structure and the majority of candidate loci were associated with bottom temperature (Figure S1 and Table S2). Overall, our filtering resulted in 1,137 loci across 232 individuals for analysis.

### 2.5 Identifying population structure

We used three approaches to investigate spatial genetic structure in our summer flounder dataset: principal component analysis (PCA) followed by two analyses using Bayesian clustering algorithms implemented in the STRUCTURE and *Geneland* programs. We performed PCA using the *adegenet* v. 2.0.0 (Jombart, 2008) and *ade4* v.1.7‐2 packages (Chessel, Dufour, & Thioulouse, 2004) in R version 3.2.2 (R Core Team 2015), and any missing data (0.57%) were interpolated using mean allele frequency.

Next, we used STRUCTURE v.2.3.4, a Bayesian clustering algorithm that is not dependent on location information (Pritchard, Stephens, & Donnelly, 2000), to identify the number of putative populations. In STRUCTURE, we used a burn‐in of 10,000 iterations followed by an additional 200,000 Markov chain Monte Carlo (MCMC) steps assuming admixture and correlated allele frequency models with prior sampling location information (Hubisz, Falush, Stephens, & Pritchard, 2009). We selected the admixture and correlated allele frequency models because they are considered to be the best when subtle population structure is expected (Falush, Stephens, & Pritchard, 2003). We initially analyzed all available 1,137 loci, and then subsequently analyzed the 1,005 that remained after removing loci not in Hardy–Weinberg proportions (HWP; *p *<* *0.01, exact test). Loci not in HWP were identified with the *pegas* v. 0.10 package (Paradis, 2010) in R. We ran 10 replicates of K from 1 to 5, where K is the number of population clusters, for each set of loci and checked for parameter stabilization using the burn‐in and MCMC lengths above.

To determine the optimal K, the 10 STRUCTURE replicates for each K were packaged and input into STRUCTURE HARVESTER, a web‐based program that facilitates visualization of STRUCTURE output and selection of the optimal *K* (Earl & VonHoldt, 2012). The mean likelihood values (*L*(*K*)), as well as Δ*K*, a second order rate of change of *L*(*K*) with respect to *K*, were used to determine the optimal number of populations. The Δ*K* method of determining the optimal *K* corresponds to the true value of *K* more often than the *L*(*K*) method, but it cannot evaluate scenarios when *K *=* *1, limiting its usefulness when a single population exists (Evanno, Regnaut, & Goudet, 2005). To visualize the percent assignment of each individual into the putative populations, output from STRUCTURE HARVESTER was fed into CLUMPP, a program that averages the mean of the posteriors from multiple runs of the optimal K (Jakobsson & Rosenberg, 2007). At last, these averaged posterior means were plotted using Distruct (Rosenberg, 2004).

As different Bayesian clustering methods can produce different results (Frantz, Cellina, Krier, Schley, & Burke, 2009), we also used the *Geneland* v.4.0.7 package in R (Guillot, Mortier, & Estoup, 2005) to avoid drawing conclusions with potentially important conservation implications on a single analysis. *Geneland* is a Bayesian clustering program that utilizes geo‐referenced multi‐locus genetic data to determine population structure. Latitude and longitude were converted to UTM and standardized to zone 18. With the full dataset of 1,137 loci in 232 individuals, we used 100,000 MCMC iterations thinned to every 100th iteration, allowed the number of K clusters to vary between 1 and 10 and applied the spatial method with the uncorrelated frequency model. We then performed an additional analysis using the correlated frequency model and examined how the posterior distribution changed (as suggested by the *Geneland* manual).

### 2.6 Isolation by distance

Pairwise genetic differences among 232 individuals using 1,137 loci were calculated using Rousset's distance (Rousset, 2000) in SPAGEDI (Hardy & Vekemans, 2002):

$$a_{r}=\frac{Q_{w}-Q_{r}}{1-Q_{w}}$$

where Rousset's distance (*a*_*r*_) is the difference between the probability of identity of two genes within an individual (*Q*_*w*_) and the probability of identity of two genes at some geographic distance *r* (*Q*_*r*_), divided by the probability of two genes not being identical within an individual. A corresponding matrix of geographic differences between individuals was calculated using least cost path analysis constrained to the continental shelf at the 200‐meter isobath in the *marmap v*.0.9.6 package (Pante & Simon‐Bouhet, 2013) in R.

A Mantel test was performed to compare pairwise genetic distances against geographic distances. The Mantel analysis was conducted with the R package *ade4* v.1.7‐2 (Chessel et al., 2004) using 10,000 permutations to test for correlation between Rousset's genetic distance and geographic distance in kilometers.

### 2.7 Estimating dispersal

To better translate our genetic structure data into inferences about ecological processes, we estimated the dispersal rate across Cape Hatteras, North Carolina, a putative zoogeographic barrier for this species, using Approximate Bayesian Computation (ABC). ABC is a powerful statistical framework that can be used to estimate demographic and evolutionary parameters from large genomic datasets without explicitly calculating a likelihood function (Tavaré, Balding, Griffiths, & Donnelly, 1997; Beaumont, Zhang, & Balding, 2002 and reviews by Bertorelle, Benazzo, & Mona, 2010; Csilléry, Blum, Gaggiotti, & François, 2010). In brief, a large number of simulations are performed, with parameters being drawn from a probability distribution. The simulated genetic data are then reduced to summary statistics. Next, the simulated summary statistics are compared to the observed statistics and a metric of distance is calculated for each simulation. The associated demographic parameters for each simulation are then either accepted or rejected based on their distance metric, yielding simulations and associated parameters whose summary statistics most closely match those of the observed data.

To estimate dispersal across Cape Hatteras, a putative zoogeographic barrier for summer flounder, we first grouped our 232 individuals into populations occurring north (*n *=* *135) or south (*n *=* *97) of Cape Hatteras, North Carolina. We limited our ABC analysis to the 663 loci (out of 1,137) with no missing data across all individuals. We then summarized these observed genetic data by computing the joint site frequency spectrum (SFS). *Fastsimcoal2* (Excoffier, Dupanloup, Huerta‐Sánchez, Sousa, & Foll, 2013) was used to generate 100,000 coalescent simulations of a simple demographic model consisting of dispersal between two populations. Population effective sizes north and south of Cape Hatteras (POPONE and POPTWO, respectively) were drawn from log‐uniform distributions from 100 to 100,000. Dispersal between these two populations (DISP) was measured as the probability of an individual dispersing to the other population, and this probability was drawn from a uniform distribution from 0 to 0.5. As low frequency SNPs were discarded during the bioinformatics process, the observed data had a MAF cutoff of 0.043. To match that of the observed data, simulated SNPs with a MAF of < 0.043 were also removed. We then downsampled the simulated loci to 663 and computed the joint SFS for each simulation.

There is still debate about how many summary statistics are appropriate for ABC analysis. Using too few summary statistics is likely to bias ABC estimates (Beaumont et al., 2002; Marjoram, Molitor, Plagnol, & Tavare, 2003), but highly dimensional datasets can also reduce the accuracy of estimation (Blum, 2010). We chose to reduce the dimensionality of our data with PCA. To avoid computational limits, the initial 10,000 simulations were used to define PCA axes in R using the *prcomp* function and the remaining 90,000 simulations and the observed data were projected onto these PCA axes. We examined the robustness of ABC parameter estimates based on the number of summary statistics used and found that the number of summary statistics employed had little effect on dispersal parameter estimation (Table S3). As we wished to maximize the proportion of variance explained in the data, we present results that retained all 10,000 principal components as summary statistics in the ABC analysis.

Approximate Bayesian Computation model selection was performed using the *abc* package (Csilléry, François, & Blum, 2012) in R. Using simple rejection sampling, we accepted the 500 (0.5%) simulations with the shortest Euclidean distance between the simulated and observed summary statistics (the PCA‐projected SFS). Using the *density* function in R, we plotted the posterior probability distributions comprised of these retained simulations for each parameter. The POPONE and POPTWO parameters were log_10_‐transformed for plotting. This allowed us to estimate our parameters of interest (POPONE, POPTWO and DISP) and to determine the degree to which summer flounder were panmictic.

### 2.8 Environmental associations using BayEnv 2.0 and redundancy analysis

To examine the possibility of locally divergent selection in summer flounder across the species range, we used two methods to identify correlations between allele frequencies and each of four environmental variables: distance along the coast, depth of sampling location, bottom temperature, and bottom salinity. The first method was a univariate Bayesian method by Coop, Witonsky, Di Rienzo, and Pritchard (2010) implemented in BayEnv 2.0 (Günther & Coop, 2013). Unlike Fst outlier tests, BayEnv 2.0 does not assume samples are evolutionarily independent, which can help account for shared demographic history. To do this, a background covariance matrix is calculated to help control for nonindependence between closely related populations. As a result, BayEnv 2.0 is more robust at determining genetic loci under selection in isolation by distance and range expansion scenarios (Lotterhos & Whitlock, 2014). Next, a Bayes factor (BF) is estimated for each locus as a measure of association strength between allele frequency at each locus and an environmental variable while accounting for the background covariance.

We used the same 232 summer flounder as for the ABC analysis, but divided individuals into five groups for BayEnv 2.0 analysis based on the following geographic boundaries: Atlantis Canyon, Hudson Canyon, Cape Hatteras, and Cape Fear (Figure 1). These geographic features were hypothesized as possible limits to dispersal of summer flounder. This division resulted in *n *=* *40, 54, 41, 60, and 37 individuals in each group, respectively, from north to south. Distance along the coast (Figure 1) was calculated for each individual using the “Law of cosines” great‐circle distance in the GEOSPHERE v.1.5‐1 package (Hijmans, 2015) in R. Environmental variables for each individual within a group were averaged and then standardized across populations, as recommended by Coop et al. (2010). For individual fish for which environmental variables were lacking, environmental data from nearby NOAA trawls occurring during a similar time period were used.

To create the background covariance matrix, loci not in HWP according to the *pegas* v.0.10 package (Paradis, 2010) in R were first removed (*p *<* *0.01, exact test), leaving 1,005 loci with which to estimate covariance between populations. BayEnv 2.0 estimates the covariance matrix using 100,000 MCMC iterations, thinned to every 500th step. The covariance matrices from the last 40 thinned MCMC iterations were averaged to create the background covariance matrix.

All 1,137 loci were examined separately for associations with standardized environmental variables using 500,000 iterations for each locus. BayEnv 2.0 was run 10 times to check for consistency between independent runs, as MCMC methods can be sensitive to initial conditions (Blair, Granka, & Feldman, 2014). The median Bayes factors (BFs) across the 10 runs were computed for each variable, with median BFs > 3 considered as suggestive of divergent environmental selection (Kass & Raftery, 1995). The significance of locus–environment associations identified by BayEnv 2.0 was tested using 10 random permutations of the environmental data. Each permuted environmental dataset was averaged and then standardized across the original BayEnv 2.0 populations. A BayEnv 2.0 analysis was then conducted using each environmental dataset and the median BF across 10 runs for each environmental variable was calculated. We then averaged the median BFs for each locus across the 10 permutations to obtain the number of BFs > 3 that would be expected under this null model of no association.

The second method used to identify potential loci experiencing environmental selection was redundancy analysis (RDA), a multivariate, ordination‐based method that summarizes the variation in a set of response variables that can be explained by a set of explanatory variables. In this case, allele frequencies comprise the response variables and the environmental data are the explanatory variables. Forester, Jones, Joost, Landguth, and Lasky (2016) used simulations to demonstrate that multivariate methods have high power to detect locally adapted loci and that these methods maintain low false‐positive rates under a variety of scenarios with differing amounts of dispersal, selection, and habitat patchiness. To perform RDA, we used the *vegan* 2.4‐1 package (Oksanen et al., 2016) in R on standardized environmental data and a centered allele frequency dataset containing only a single allele at each of 1,137 loci across our 232 summer flounder adults. Similar to PCA, any missing data were interpolated using mean allele frequency. Potential outlier loci were identified as loci with scores ± 3 standard deviations from the mean axis score for each of the first three constrained axes. Potential outlier loci were then tested for association with environmental variables by regressing allele frequencies against each environmental variable and calculating the correlation coefficient. We set the cutoff for a significant relationship at *p*‐value < 0.001. To test the significance of locus–environment associations identified by RDA, we randomized the environmental data 10,000 times and identified outliers and associations with the environment as described above. In addition, we relaxed the assumption of linearity by fitting generalized additive models (GAMs) between the RDA outlier loci and each environmental variable. We looked for significant locus–environment associations (*p*‐value < 0.001) in the same way as for the linear models.

We used BLAST annotation to explore the gene function of contigs containing environmentally associated loci from both the BayEnv 2.0 and RDA analyses using the megablast and blastx algorithms with the nucleotide collection (nr/nt) and nonredundant protein sequences (nr) databases, respectively. We also used the megablast algorithm against the *Paralichthys olivaceus* genome.

## 3 RESULTS

### 3.1 Genotyping results

Sequencing of 280 individuals resulted in a total of nearly 158 million reads. Demultiplexing of raw reads using Illumina indices resulted in 143.1 million reads. The average number of quality filtered reads per individual was 511,179 ± 294,522 reads (mean ± standard deviation). The dDocent pipeline identified 89,549 unique contigs, with an average per nucleotide read depth greater than 39X. Contig sequences with ≥90% similarity were clustered together, resulting in a de novo reference assembly of 46,894 contigs. From this, 313,477 putative SNPs were identified throughout the summer flounder genome. After applying our different filtering steps, 1,137 highly informative loci across 232 individuals were retained for subsequent analysis.

### 3.2 Identifying population structure

**Figure 2:**
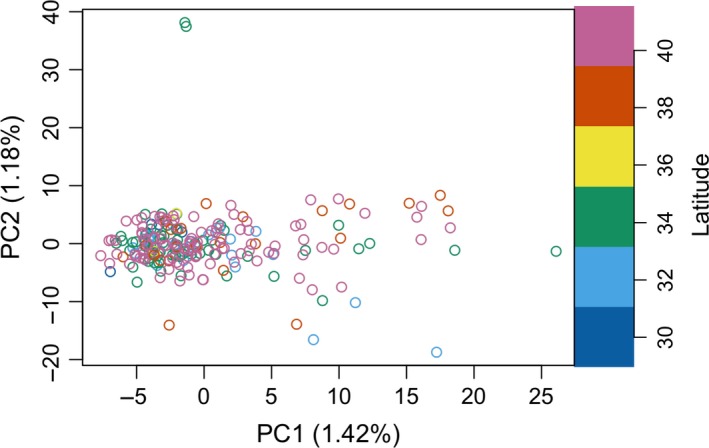
Principal component analysis plot of 232 individuals at 1,137 loci. Color indicates latitude

The PCA of 1,137 loci sampled from 232 summer flounder adult individuals revealed no distinctive population structure along the U.S. east coast between Massachusetts and Florida (Figure 2). The first and second principal components explained 1.42% and 1.18% of the variance, respectively.

**Figure 3:**
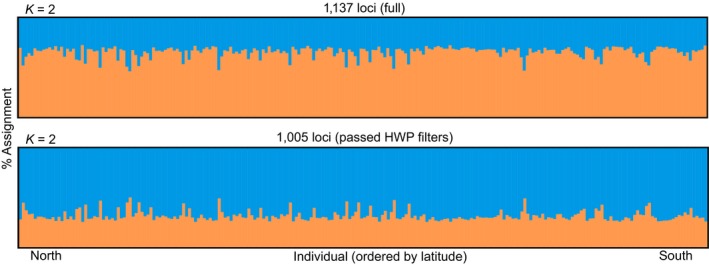
Bayesian clustering assignment implemented in STRUCTURE using the full dataset of 1,137 loci (upper panel) and 1,005 loci passing HWP filters (lower panel), with colors indicating clusters, or populations. Using all 1,137 loci, the optimal number of clusters was *K *=* *2 (upper panel). The optimal number of clusters using 1,005 loci that passed HWP filters was *K *=* *2 (lower panel). Taken together, we interpret these analyses as a lack of population structure in summer flounder

Analysis in STRUCTURE revealed minimal population structure, with no clear geographic boundaries. After testing *K *=* *1 to *K *=* *5, both the mean likelihood values (*L*(*K*)) and the Evanno method (Δ*K*) suggested two clusters (*K *=* *2) regardless of the set of loci (all 1,137 or 1,005 passing HWP filters) used in the analysis (Figures 3 and S2). We initially used all available 1,137 loci to assign 232 individuals to populations. We did this to avoid prematurely removing loci potentially contributing to population structure. We then removed 132 loci not in HWP and performed an additional STRUCTURE analysis using the 1,005 loci that passed HWP filters, as is more typical of population assignment analyses. Even though *K *=* *2 using the full dataset of 1,137 loci and the subset of 1,005 loci that passed HWP filters, we interpreted these STRUCTURE analyses as a lack of population structure in summer flounder because individuals did not group into separate populations, but were instead admixed at approximately the same proportions.

*Geneland* analysis using the uncorrelated frequency model indicated greatest posterior density at one cluster (*K *=* *1). The correlated frequency model in *Geneland* is better at detecting subtle population structure, but is more prone to instability and sensitive to departure from model assumptions. The correlated frequency model had greatest posterior density at *K *=* *10, but clustering was not geographically related. In comparison with the uncorrelated frequency model, we interpreted the correlated frequency model results as model overfitting due to the limitations of the correlated frequency model. Thus, we conclude that *Geneland* analyses also suggest a single population in summer flounder (Figure S3).

### 3.3 Isolation by distance

**Figure 4:**
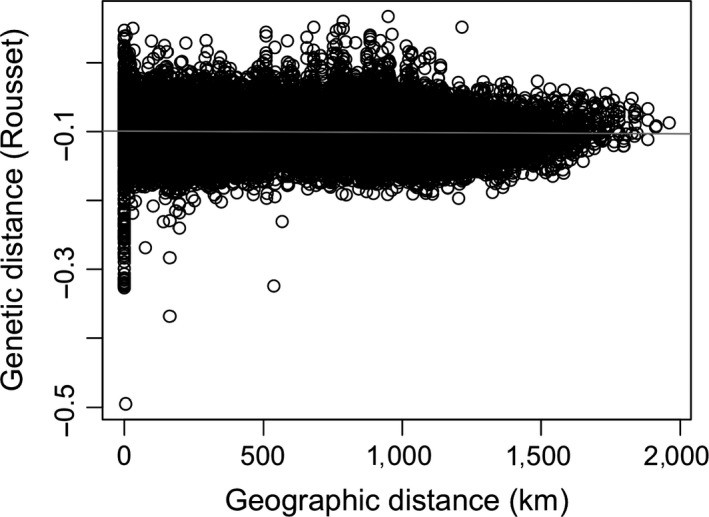
Isolation by distance of 232 individuals using Rousset's distance for genetic distance and least cost path analysis constrained to the continental shelf for geographic distance. Pairwise comparisons of individuals captured in the same tow have geographic distances of zero

We found no detectable relationship between geographic distance and genetic distance, as measured by Rousset's distance (*a*_*r*_), across 232 individuals genotyped at 1,137 loci (*r *=* *0.026; Mantel test *p *>* *0.86; Figure 4). Distant summer flounder were as genetically similar as those nearby to each other.

### 3.4 Estimating dispersal

**Figure 5:**
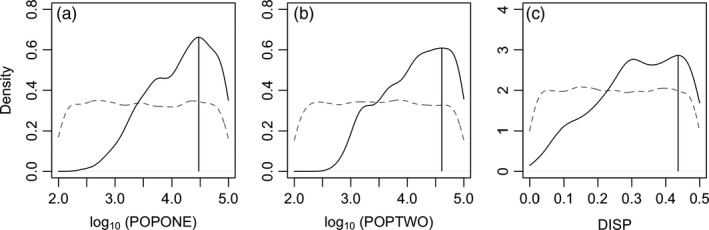
Posterior probability distributions of (a) effective population size north of Cape Hatteras (POPONE), (b) effective population size south of Cape Hatteras (POPTWO), and (c) dispersal rate between north and south of Cape Hatteras (DISP). Prior distributions (gray dashed lines) and posterior distributions (solid black lines) demonstrate that the summary statistics were relevant to parameter estimation. Effective population size estimates are in log_10_ and vertical black lines indicate posterior modes

Approximate Bayesian Computation analysis revealed that effective population size both north and south of Cape Hatteras (POPONE & POPTWO) and the dispersal rate between these two groups (DISP) are unlikely to be small (Figure 5 and Table 2). The 95% credible interval bounds for effective population size north of Cape Hatteras were 935 and 95,126 individuals, while those south of Cape Hatteras were 1,056 and 92,491 individuals. The 95% credible interval bounds for the dispersal rate across Cape Hatteras were 0.071 and 0.496. Given the observed data, there is a 95% probability that the true dispersal rate is greater than 0.071.

**Table 2:** Prior and posterior distributions of parameters estimated using ABC analysis

Parameter	Prior	Mode	95% CI
N_e_ north of Cape Hatteras: POPONE	logunif (10, 100,000)	29,888	935–95,126
N_e_ south of Cape Hatteras: POPTWO	logunif (10, 100,000)	40,710	1056–92,491
Dispersal rate between populations: DISP	unif (0, 0.5)	0.43	0.071–0.496

### 3.5 Environmental associations

Our summer flounder samples were distributed over a wide geographic area, characterized by gradients of environmental variables (Figures S4 and S5). BayEnv 2.0 analyses revealed that 14 of 1,137 loci (Table 3; Figure S6) had strong positive associations with at least one environmental variable (median BF > 3). In total, bottom temperature was correlated with 10 loci, the greatest number for any environmental variable tested. Distance along the coast was correlated with nine loci, and both depth and bottom salinity with six loci. Analysis of randomized environmental data in BayEnv 2.0 identified that our null expectation should be five loci associated with depth, four with bottom salinity, two with bottom temperature, and one with distance. For each environmental variable, we found the empirical cumulative observed distribution of BFs to be significantly different than the permuted distribution (two‐sample K‐S test; *p *<* *0.00001; Figure S7). We also binned BFs to determine if the observed number of BFs > 3 was greater than would be predicted by chance alone for each environmental variable (Figure S8). There were significant differences between the number of BFs > 3 for distance and for bottom temperature (binomial exact test, *p *<* *0.0001), but no significant differences for depth or for salinity (binomial exact test, *p *>* *0.05). Regarding these differences, there were both a greater number of high locus–environment *R* ^2^ values and a greater proportion of significant BFs by chance for some environmental variables, and this tended to occur for environmental variables with fewer unique values (depth and bottom salinity). The standard error of the expected proportion of significant BFs decreased with increasing number of unique environmental values, resulting in greater power to detect nonrandom locus–environment associations for variables with larger numbers of unique values (distance and bottom temperature; Figure S9).

**Table 3:** Median Bayes factors (BF) for locus–environment associations suggestive of local adaptation (BF > 3) after ten independent BayEnv 2.0 runs. BF values are printed

Environmental variables					
Contig number	Variant BP	Distance along the coast	Depth	Bottom temperature	Bottom salinity
2558	83				3.84
8420	14	8.85	6.85	6.9	
15075	20	8.75	6.41	10.59	
27738	24	3.49	3.65	3.41	
31661	32	4.58		5.04	
35399	131	3.11		3.82	
37577	42			3.25	
38595	30				3.68
42485	39	4.16		3.35	3.21
42614	134	6.84	4.79	4.44	9.6
54409	32		3.25		9.86
61273	27	4.09	5.09	3.13	
69416	44	3.14		5.11	
75053	12				3.18

**Figure 6:**
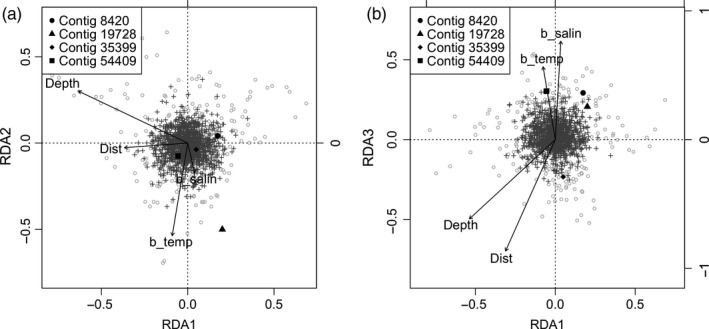
Ordination plots for RDA. Plots show the distribution of loci (gray pluses) and genotypes of individuals (gray open circles). The black vectors indicate correlations between the constraining axes and the predictor (environmental) variables. The loci (variant bp contained on a contig) whose allele frequencies are significantly (*p *<* *0.001) associated with one or more predictor variables are plotted on (a) axis 1 vs. 2 and (b) axis 1 vs. 3

RDA identified 23 outlier loci, of which four were significantly (*p *<* *0.001) associated with one or more environmental variables (Figure 6 and Table 4). Five locus–environment associations were detected: bottom salinity was correlated with two loci, and bottom temperature, distance and depth were each associated with one. The GAMs identified the same five locus–environment associations (Figure S10). Three of the four spatially divergent RDA candidate loci also emerged as candidates in the BayEnv 2.0 analysis (contigs 8420, 35399 and 54409). Although the total number of RDA outliers we identified was to be expected under a null model (*p *=* *0.362; Figure S11A), the number significantly associated with an environmental variable was not. We identified five significant locus–environment associations using RDA and regression, and this was unlikely to occur under a null model (*p *=* *0.044; Figure S11B).

**Table 4:** P‐values of locus–environment associations suggestive of local adaptation from redundancy analysis and regression (*p*‐value <0.001)

Environmental variables					
Contig number	Variant BP	Distance along the coast	Depth	Bottom temperature	Bottom salinity
8420	14				1.49 × 10^−4^
19728	116		2.77 × 10^−5^	2.62 × 10^−5^	
35399	131	5.98 × 10^−04^			
54409	32				6.09 × 10^−4^

Using BayEnv 2.0 and RDA, we identified a total of 15 loci whose allele frequencies were regionally differentiated and strongly associated with environmental variables (Table S4). BLAST annotation of these 15 candidate loci using the megablast and blastx algorithms revealed several strong matches (*e*‐value ≤ 1E‐4) with fish species (Table S5). Six of the candidate loci with strong matches aligned closely with the olive flounder (*Paralichthys olivaceus*) genome and have putative functions associated with calcium binding, metal binding, demethylation, transcription, and transmembrane proteins. BLAST annotation against the *Paralichthys olivaceus* genome also returned many annotated genes that were either embedded within or adjacent to candidate loci (Table S5).

## 4 DISCUSSION

Marine populations were often assumed to be homogenous because there are few apparent barriers to dispersal, but recent evidence instead suggests that dispersal is often highly constrained. Using 1,137 loci genotyped in 232 adult summer flounder along nearly the entire U.S. east coast, we found that these fish comprise a single ecological population with no indication of isolation by distance. However, summer flounder reside across wide environmental gradients, and we also found genomic signatures suggestive of divergent environmental selection on regional scales, despite sequencing only a small part of the genome. In particular, we found 15 loci whose allele frequencies were associated with at least one environmental variable, and 11 (73%) of them were correlated with bottom temperature.

### 4.1 Population differentiation

Renewed interest in understanding summer flounder population biology in light of shifts in biomass (Bell et al., 2014; Nye et al., 2009; Pinsky & Fogarty, 2012) provides strong motivation for the application of molecular techniques to fisheries‐relevant research. From PCA, STRUCTURE, and *Geneland* analyses, we conclude that summer flounder are a genetically homogenous population across much of the genome. Even if some of our sampled individuals were immature, we expect population structure would be detected because juveniles and adults move offshore together during the spawning season (Packer et al., 1999). A naïve interpretation of our STRUCTURE analyses would suggest multiple populations, but inconsistencies in the number of clusters inferred by different programs can occur (Frantz et al., 2009) and it is important to interpret results with biological relevance in mind (Meirmans, 2015). In addition, while the correlated allele frequency models implemented in STRUCTURE and *Geneland* can help distinguish between closely related populations, this also makes them more prone to overestimating K (Falush et al., 2003; Pritchard et al., 2000). Using ABC analysis to examine population differentiation from another perspective, we estimated the dispersal rate across Cape Hatteras, North Carolina. Cape Hatteras has been suggested as an important dispersal barrier in this species (Able et al., 2011; Burke et al., 2000; Kraus & Musick, 2000; Van Housen, 1984). However, the ABC analysis suggested that the probability of an individual dispersing across this putative barrier was not low, and in fact, was likely quite high (7% to 50% probability per generation). Dispersal rates of ~10% or greater are often suggested as sufficient to synchronize population dynamics (Hastings, 1993), and a population can be defined under an ecological paradigm by focusing on demographic connectivity (Waples & Gaggiotti, 2006). Thus, the dispersal rates that we estimated would imply that summer flounder north and south of Cape Hatteras comprise one population from an ecological and population dynamics perspective.

Previous studies investigating population structure in summer flounder using genetic techniques (Jones & Quattro, 1999; Van Housen, 1984) or phenotypic traits (Able et al., 1990, 2011; Burke et al., 2000; Kraus & Musick, 2000) have disagreed on the existence of subpopulations. Our genetic analyses with 1,137 SNPs had higher statistical power than earlier mtDNA analyses (Jones & Quattro), but reached the same general conclusion that summer flounder exhibit extensive gene flow across the entire U.S. east coast. Earlier observations of differences in the timing of larval ingress (Able et al., 2011), morphology, and meristics (Burke et al., 2000) suggest that summer flounder may respond plastically to environmental differences across the species range, resulting in differences in phenotype despite extensive gene flow. An alternative hypothesis is that differences in phenotype are controlled or linked to loci under selection, potentially some of the loci identified by this study that were associated with an environmental variable. A productive avenue for future research would be to employ common garden studies to determine the underlying basis of phenotypic variation in this species.

### 4.2 Evidence for spatially divergent selection

Despite technological advances in an era of genomics, identifying adaptive divergence in natural populations is still challenging (Bernatchez, 2016). Yet, reduced genome representation techniques like ddRAD sequencing can facilitate insight into the demographic and evolutionary processes that shape the genomes of nonmodel organisms (McKinney, Larson, Seeb, & Seeb, 2017). Given that this study surveyed ~ 0.04% of the summer flounder genome (based on the 546 Mb olive flounder genome; Shao et al., 2017), it may have missed many adaptive polymorphisms (Lowry et al., 2017). In addition, recent research suggests that environmental adaptation affects a small fraction of the genome (Bay et al., 2017), and that adaptive traits are often controlled by many loci of small effect (Le Corre & Kremer, 2012; Pritchard & Di Rienzo, 2010; Rockman, 2012; Yeaman, 2015). These two factors can further complicate the identification of loci involved in adaptation. However, despite limited ability to detect polygenic adaptation and loci with small effect sizes, locus–environment associations are still useful for uncovering candidate loci of relatively large effect. Our study likely missed adaptive polymorphisms due to limited genome sampling and limited power to detect small effect loci, but we still detected a number of important candidate loci that appear to be affected by spatially varying natural selection. Furthermore, the lack of overall genetic population structure suggests that the species is locally divergent only at particular loci and that our environmentally associated loci are not the result of underlying neutral population structure. Our findings, which are suggestive of adaptive divergence in summer flounder, are consistent with a growing number of other studies in marine species that have documented adaptive polymorphisms across the species range despite high gene flow (De Wit & Palumbi, 2013; Gagnaire, Normandeau, Côté, Hansen, & Bernatchez, 2012; Gleason & Burton, 2016; Pespeni, Chan, Menge, & Palumbi, 2013; Pespeni & Palumbi, 2013; Sandoval‐Castillo, Robinson, Hart, Strain, & Beheregaray, 2018; Schmidt & Rand, 1999; Therkildsen et al., 2013).

Even though high gene flow can introduce maladaptive alleles to local populations and reduce the degree to which they can locally adapt, genetic differentiation at particular loci can still arise if selection is strong enough (Hedgecock, 1986; Marshall, Monro, Bode, Keough, & Swearer, 2010; Slatkin, 1987). Selection can more effectively filter out maladaptive alleles or increase the frequency of beneficial alleles when the effective population size is large and thus genetic drift is weaker (Lanfear, Kokko, & Eyre‐Walker, 2014; Ohta, 1992), as was suggested for summer flounder by our ABC analysis. The process of strong selection on a local scale following widespread dispersal can result in the maintenance of adaptive polymorphisms. These polymorphisms are maintained through spatial balancing (also called spatially varying) selection and have been termed spatially balanced polymorphisms (Sotka, 2005, 2012). In an alternative way, low dispersal or active habitat selection can also drive genetic differentiation across space (Kawecki & Ebert, 2004), which is often known as local adaptation. Although the scale of gene flow and selection differ between local adaptation and spatial balancing selection, they lie on a continuum and both result in genetic differences between local sites (Sanford & Kelly, 2011). In the case of summer flounder, selection following dispersal in each generation appears to be the most likely process maintaining genetic differentiation at certain loci. Evidence for locally adapted polymorphisms maintained by spatial balancing selection in summer flounder can be strengthened by comparing allele frequencies in larvae to those in adults, which provides a promising avenue for future research. If allele frequencies at a set of loci are homogenous in larvae, but divergent in adults, this would suggest that genetic differentiation in adults is maintained by spatial balancing selection.

Processes other than spatial balancing selection could result in polymorphisms throughout the genome, but we believe these are less likely in the summer flounder system. One factor that can drive differentiation at particular loci is sex‐specific genetic markers. Phenotypic sex in summer flounder is genetically determined through male heterogamety, or a XX/XY sex‐determining system (Colburn, 2008). We did not find any SNPs in our dataset that were present in all males and completely absent in females, as would be expected of male‐specific markers in a male heterogeneous species. However, our ability to identify such markers in our dataset is somewhat limited. Studies explicitly seeking to identify sex‐specific genetic markers typically aim to understand genetic sex determination and the evolution of sex‐determining systems (Carmichael et al., 2013; Fowler & Buonaccorsi, 2016; Gamble & Zarkower, 2014). As a result, these studies undertake bioinformatics steps to limit RAD tags to those found in only one phenotypic sex and not the other (which we did not do). In addition, ~20% of individuals were not confidently sexed in our dataset, and we did not aim to explicitly identify sex‐linked markers. Despite these caveats, it seems unlikely that any sex‐linked markers were retained in our final dataset. Demographic processes like range expansion and reproductive sweepstakes can also result in spatial differences at a few loci. Recent range expansion can bring alleles into newly colonized areas, resulting in allele frequency gradients due to genetic drift as the expansion front moves across the landscape. This process is termed allelic surfing and can result in patterns at neutral loci that resemble signatures of selection (Excoffier & Ray, 2008; Hallatschek, Hersen, Ramanathan, & Nelson, 2007). However, allelic surfing is more likely to happen with low gene flow (Excoffier & Ray, 2008; Klopfstein, Currat, & Excoffier, 2006), which makes it unlikely in summer flounder because of high dispersal estimates and no recent history of range expansion. High reproductive variability in broadcast spawners, such as summer flounder, can also create temporal patterns of genetic differentiation (Hedgecock & Pudovkin, 2011). This process is mostly applicable to benthic adult populations and can result in transient “chaotic patchiness,” or chaotic patterns of genetic divergence across space in each time step. While adult summer flounder are capable of movement, our candidate loci were associated with environmental gradients rather than exhibiting chaotic patterns typical of sweepstakes reproduction. Instead, we suggest that spatially varying selection due to environmental heterogeneity along the coastline is maintaining adaptive divergence at particular loci in summer flounder, but further investigation is necessary to strengthen the evidence for this.

Environmental conditions can be important selective agents that shape the genotypic and phenotypic composition of local populations (Sanford & Kelly, 2011). Water temperature is perhaps the most important abiotic factor defining a marine organism's habitat (Angilletta, 2009). For many ectotherms, the temperature of their surrounding environment controls a variety of physiological processes, including aerobic scope (Pörtner & Knust, 2007) and developmental (O'Connor et al., 2007) and metabolic rates (Johnston & Dunn, 1987), which in turn can affect growth and survival. In a classic example, Powers and Schulte (1998) summarize the genomic and phenotypic evidence for thermal adaptation in the mummichog *Fundulus heteroclitus* along the Atlantic coast of North America. In particular, the steep thermal cline associated with latitude was tightly linked with the frequency of lactate dehydrogenase allozymes. In turn, the frequency of lactate dehydrogenase allozymes was correlated with ATP concentration and oxygen affinity in red blood cells, which affected swimming performance at cool temperatures. In the context of summer flounder, an improved understanding for why certain loci are associated with environmental variables, such as identifying functional relationships between environmental selective agents and locus‐specific selection, will strengthen the evidence for spatially divergent selection. While some of our BLAST searches returned orthologous matches with other fish species, summer flounder is a nonmodel organism and it was challenging to draw robust conclusions about gene function with the available resources. However, whole‐genome sequencing of a closely related species, the olive flounder, or Japanese flounder (*Paralichthys olivaceus*), was recently completed (Shao et al., 2017) as part of the 1,000 Plant & Animal Reference Genomes Project, and several linkage maps using nuclear markers are available (Castaño‐Sánchez et al., 2010; Kang, Kim, & Lee, 2008; Shao et al., 2015). Our candidate loci could have important gene functions that have yet to be annotated, or they may be physically linked to other selected loci that we did not sample. Regardless, further annotation of the olive flounder genome, as well as those of other fish species, would allow for a more complete understanding of linkage, gene function, and why our candidate loci may be important indicators of selection in summer flounder.

### 4.3 Potential for adaptation in a changing world

Within the context of global environmental change, spatial balancing selection can allow for all phenotypes and their corresponding genotypes to be present in populations across the species range. Selection acts on the local scale to filter for phenotypes and genotypes that are best suited to local conditions. As local conditions shift over time, different phenotypes and their underlying genotypes may become more or less favorable depending on current conditions. The ability of selection to match phenotype with the current environmental conditions results in a high potential for adaptation as the environment changes (Sanford & Kelly, 2011). Of course, adaptive potential also depends on the underlying genetic variation in a species. Historical studies of exploited fish stocks have shown that fishery‐induced population declines can lower effective population size (N_e_) and erode standing genetic variation (Hauser, Adcock, Smith, Ramiréz, & Carvalho, 2002; Hutchinson, van Oosterhout, Rogers, & Carvalho, 2003; Pinsky & Palumbi, 2014). If existing genetic variation and effective population size are low in summer flounder due to historical fishing pressure, adaptive evolution in response to changing environments may be limited. Future studies investigating the relationship between standing genetic diversity and the effectiveness of selection at matching phenotypes and genotypes to local environmental conditions would contribute toward a better understanding of evolutionary potential in response to environmental change.

In conclusion, summer flounder from Massachusetts to Florida comprise an effectively panmictic population across much of their genome. We detected genomic signatures suggestive of spatially divergent environmental selection at a few select loci, with many of these divergent loci associated with bottom temperature. These loci are good candidates for further investigation into their functional role in adaptation to changing environmental conditions, and they appear to be maintained by selection rather than limited gene flow. Understanding the relevance and balance between dispersal potential, the strength of selection, and the scale of environmental heterogeneity can help us to understand the adaptive potential and persistence of summer flounder and other important exploited species under future climate conditions.

## CONFLICT OF INTEREST

None declared.

## DATA ARCHIVING STATEMENT

Raw sequencing reads have been archived in the NCBI Sequence Read Archive (SRA) database (Acc. No. SRA SRP150953). Other data and files associated with this study are available at Dryad Digital Repository: https://doi.org/10.5061/dryad.c6p8q62.
