# Supplementary material for: Genomic signatures of environmental selection despite near‐panmixia in summer flounder
Source: Evol Appl. 2018 Aug 17;11(9):1732–47. doi: 10.1111/eva.12676 (PMC6183468; doi:10.1111/eva.12676)
Supplement: Supplementary file 1 [file EVA-11-1732-s001.docx]

**Genomic signatures of environmental selection despite near-panmixia in summer flounder**

Figure S1. Analyses including all 241 fish, including A) PCA, B) isolation by distance using individual relatedness and least cost path analysis constrained to the continental shelf, C) STRUCTURE assuming the admixture and correlated allele frequency models with prior sampling location information using all available 1137 loci (top panel) and then 968 loci that passed HWP filters (bottom panel) where † indicates the 10 fish that were captured in the same tow on Georges Bank, and D) *Geneland* using the spatial method with the uncorrelated frequency model. We randomly removed 9 of 10 fish from analyses presented in the main text because no other tow displayed a similar level of individual relatedness. Inclusion of these fish in analyses resulted in similar conclusions of summer flounder as a single population with no isolation by distance along the coast.


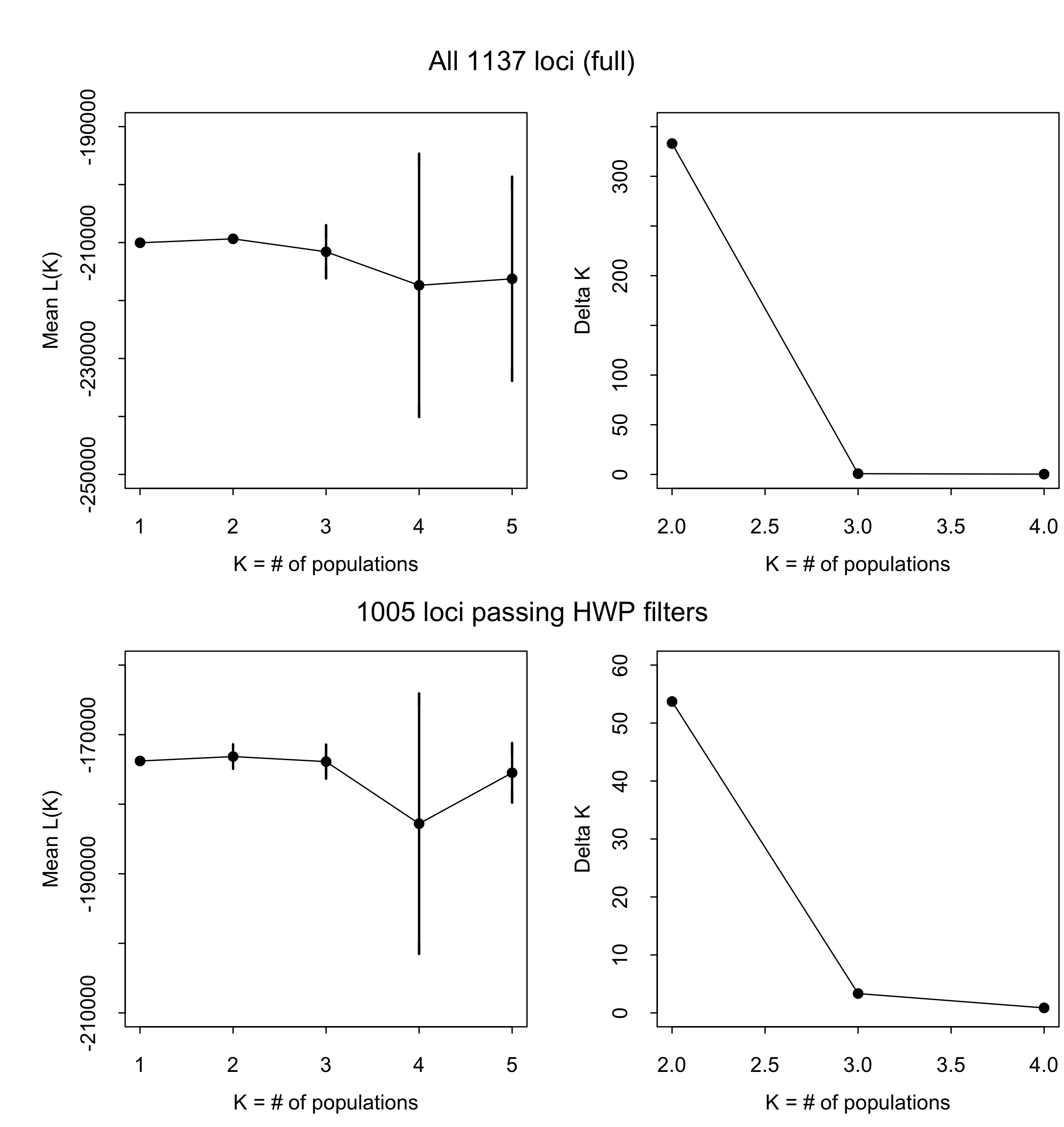


Figure S2. Mean L(K)’s, standard deviations and mean ΔK’s for K = 1 through K = 5 following 10 STRUCUTURE replicates using all 1137 loci (top panels) and using 1005 loci passing HWP filters (bottom panels).

Figure S3. *Geneland* analysis using the uncorrelated frequency model indicates greatest posterior density at one cluster (K = 1) following 100,000 MCMC iterations thinned to every 100^th^ iteration while allowing K to vary between 1 to 10. When K = 1, all individuals group into a single population (yellow box in right-most panel).


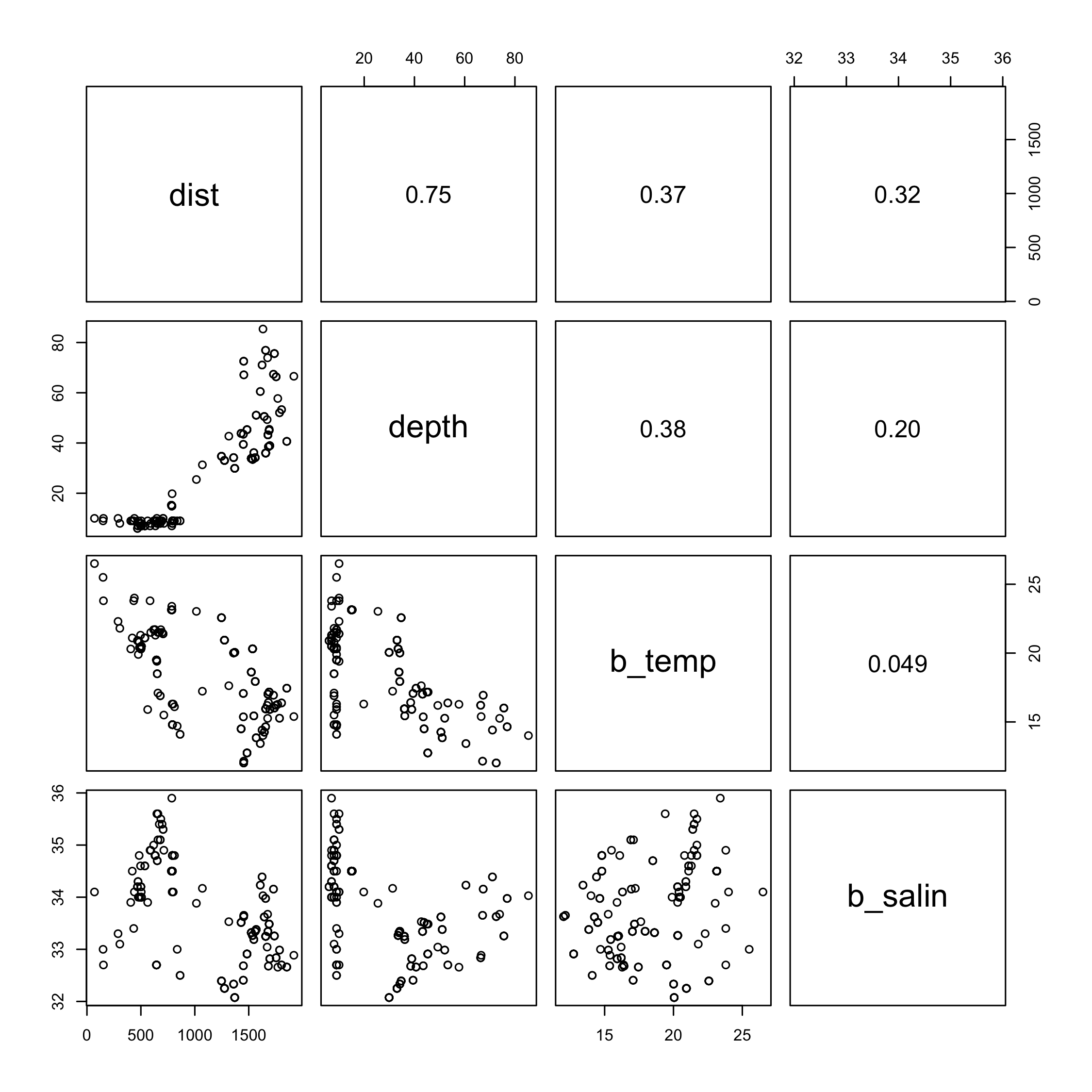


Figure S4. Pairwise scatterplot matrices and R^2^ values of the four environmental variables used to test for associations with allele frequencies. Each point corresponds to the environmental variables for an individual summer flounder. Dist = distance along the coast (kilometers), depth = depth (meters), b_temp = bottom temperature (°C) and b_salin = bottom salinity.

Figure S5. Maps of (a) bottom temperature and (b) bottom salinity where each individual summer flounder was captured.


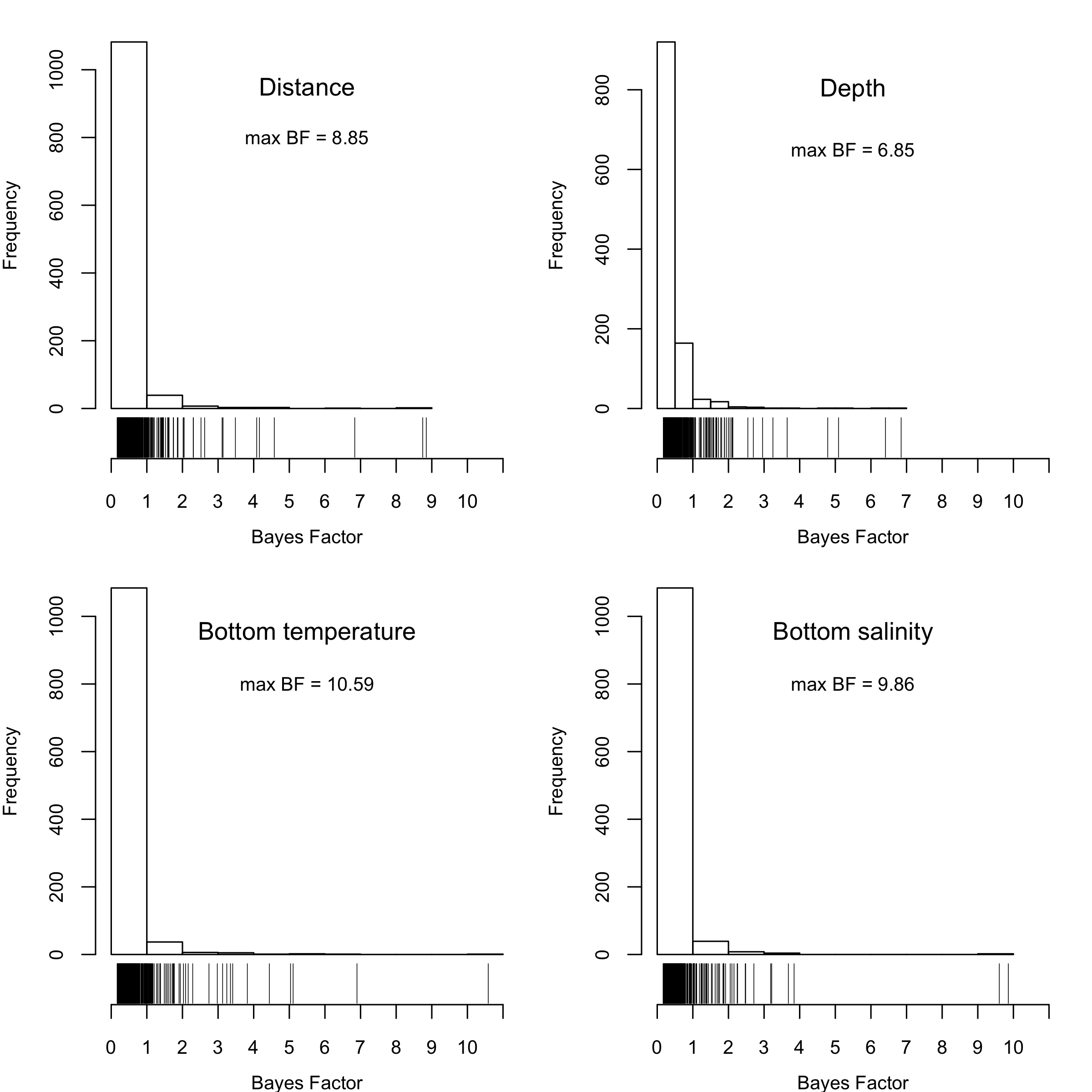


Figure S6. Median Bayes Factor (BF) for each of 1137 loci tested following 10 runs in BayEnv 2.0 for four environmental variables. Rug representation at the base of the plots indicates histogram count density.


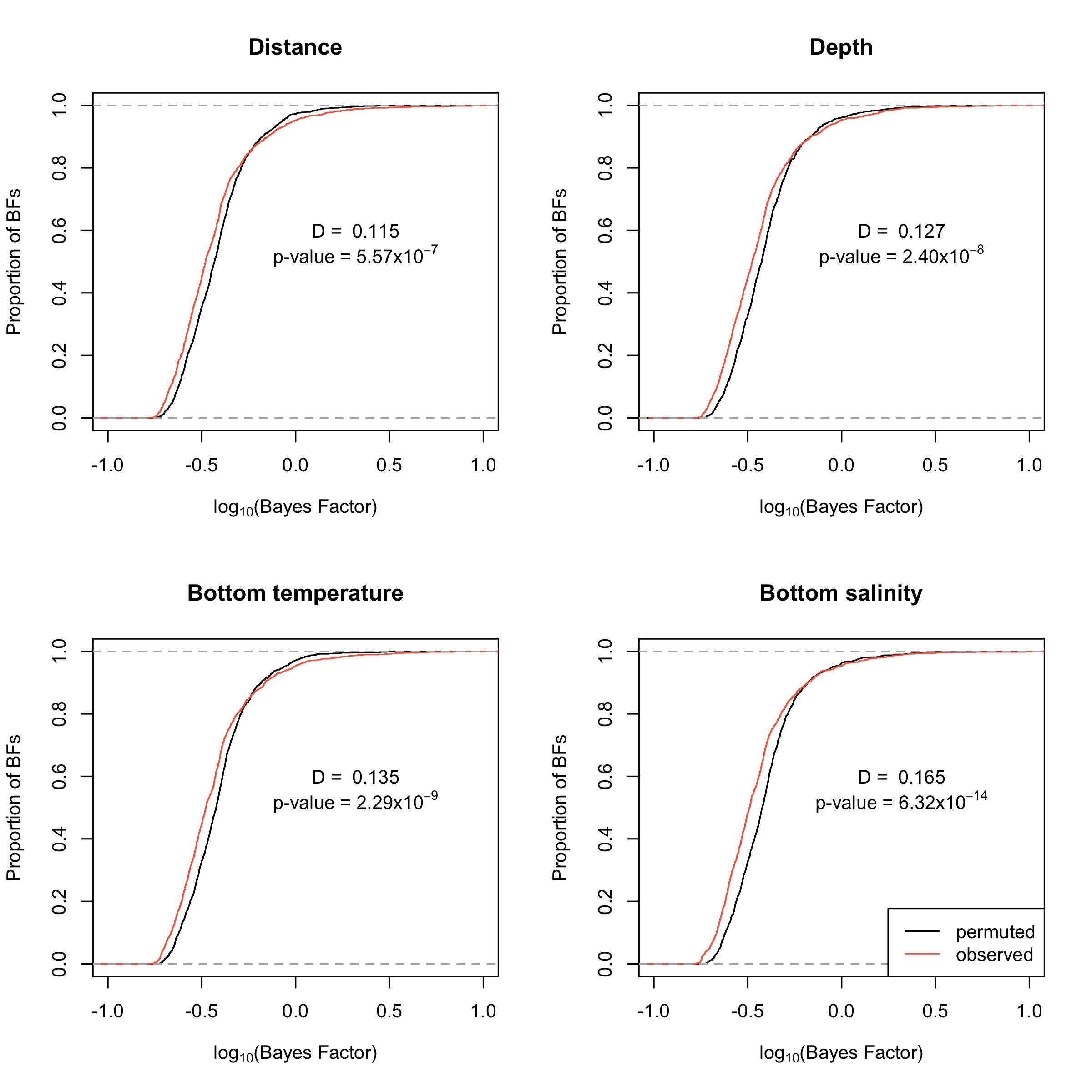


Figure S7. Empirical cumulative distribution functions of observed (red line) Bayes Factors (BFs) versus those averaged across 10 permutations (black line) for each environmental variable. BFs have been log_10_-transformed for easier viewing.


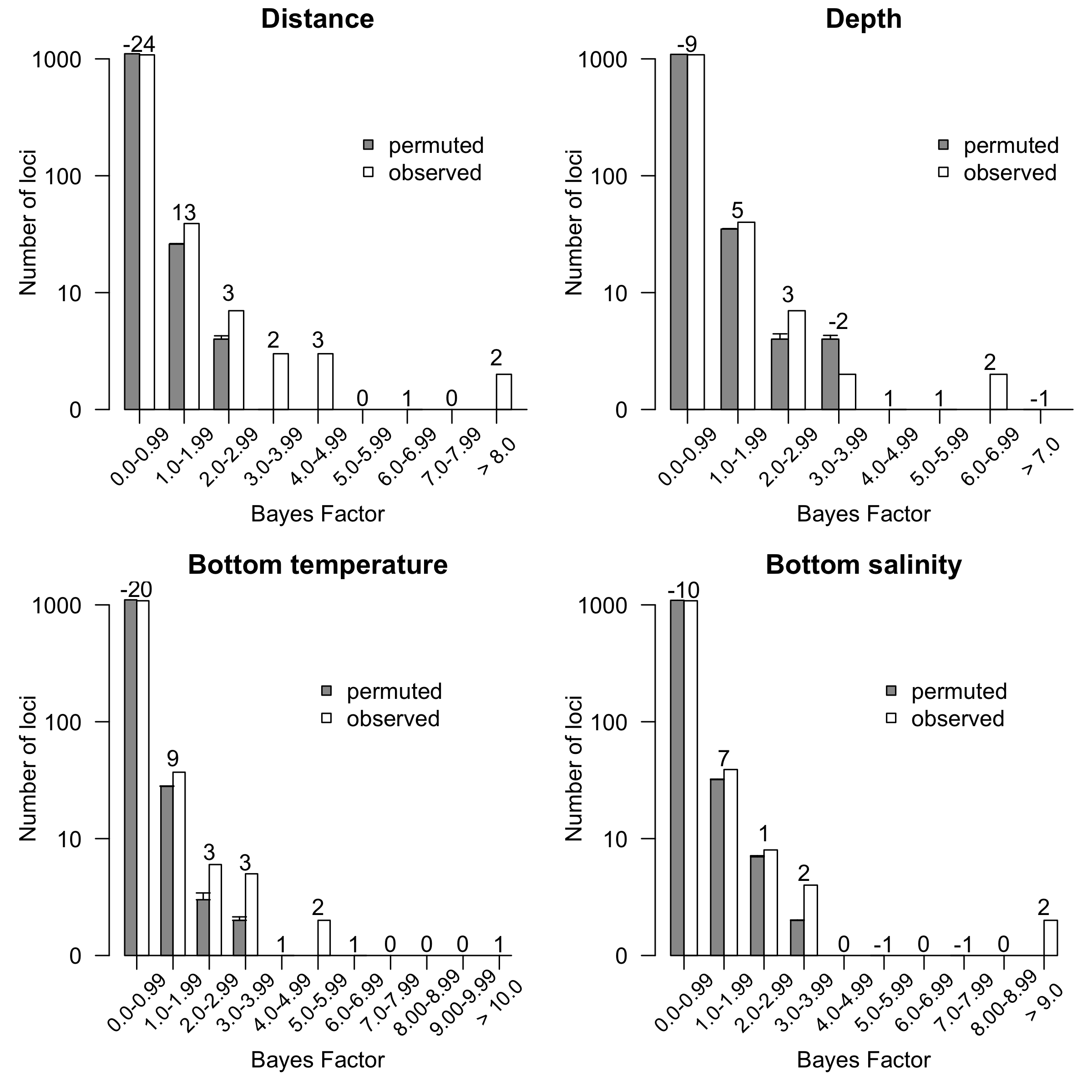


Figure S8. Distributions of observed Bayes Factors (open bars; 1137 loci) versus those averaged across 10 permutations (solid bars). Error bars represent standard deviation of permuted distributions. Numbers above the bars indicate the excess number of loci observed compared to mean permuted.


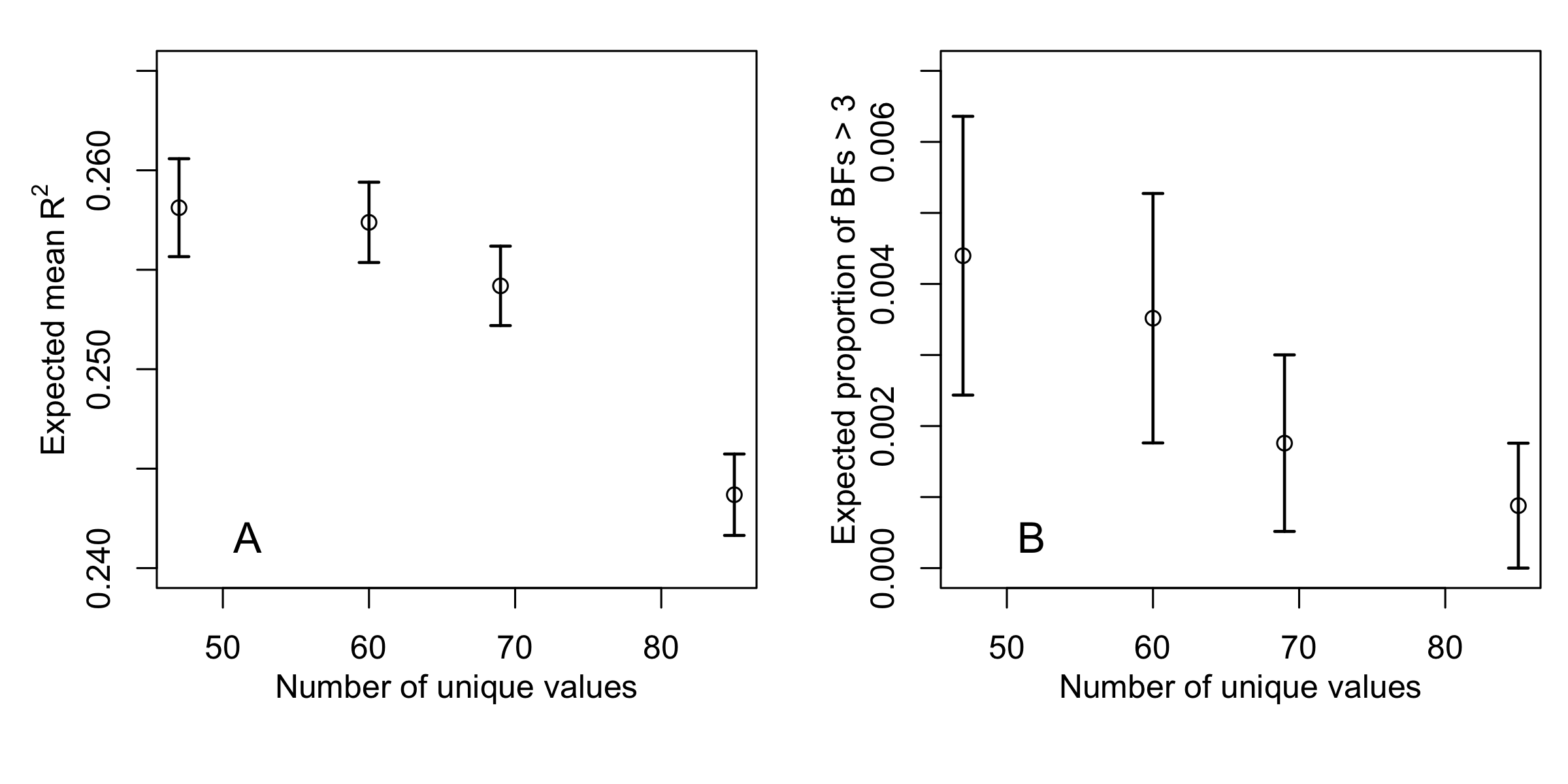


Figure S9. Expected (A) mean locus-environment R^2^ across all 1137 loci for each environmental variable and (B) proportion of BFs > 3 for each environmental variable in the null model. Error bars are standard error. Sampled environmental data for distance, bottom temperature, bottom salinity and depth consisted of 85, 69, 60 and 47 unique values, respectively.


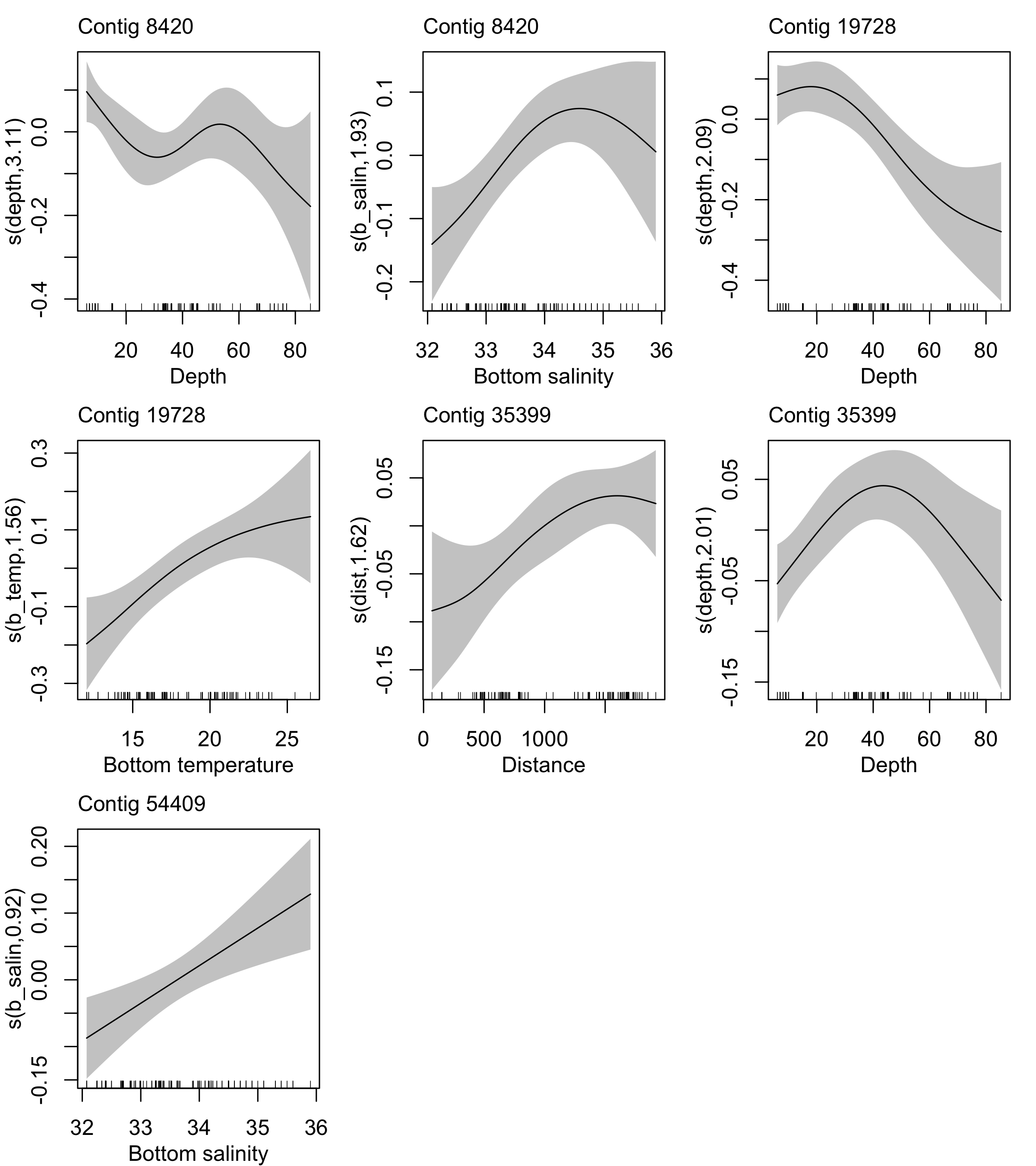


Figure S10. Generalized additive model (GAM) fits for significant associations (*P* < 0.001) between candidate loci detected from RDA and the environment. The black line in each plot illustrates the predicted allele frequency at a contig locus as a function of the environmental variable on the x-axis. The gray shaded area delimits two standard errors of the estimates. Rug along the bottom of each plot indicates density of environmental data. Units of measurement for environmental variables: Distance = kilometers, depth = meters & bottom temperature = °C.


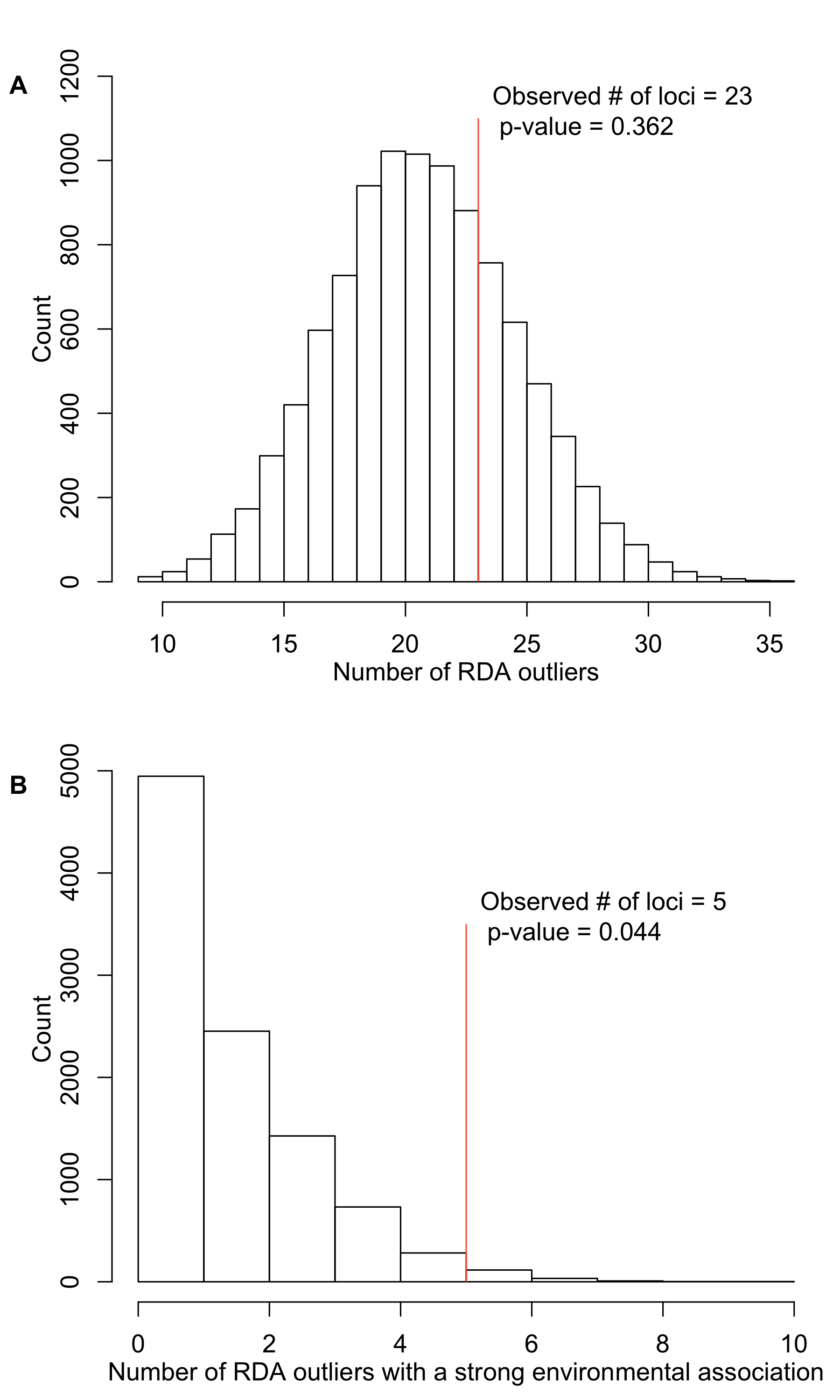


Figure S11. Randomization tests for (A) number of RDA outliers and (B) number of RDA outliers with a significant locus-environment association (*P* < 0.001). Distributions represent 10,000 randomized environmental datasets. The red lines indicate the observed number of loci for each test. The p-value represents the proportion of simulated datasets in which the number of loci was greater than the observed value.

Table S1. Multiplexing PCR primers used to amplify DNA during ddRADseq. Illumina indices within each PCR read 2 primer are underlined.

| **Primer** | **Sequence (5’ to 3’)** |
| --- | --- |
| PCR1 | AATGATACGGCGACCACCGAGATCTACACTCTTTCCCTACACGACG |
| PCR2 Illumina Index 2 | CAAGCAGAAGACGGCATACGAGATACATCGGTGACTGGAGTTCAGACGTGTGC |
| PCR2 Illumina Index 3 | CAAGCAGAAGACGGCATACGAGATGCCTAAGTGACTGGAGTTCAGACGTGTGC |
| PCR2 Illumina Index 4 | CAAGCAGAAGACGGCATACGAGATTGGTCAGTGACTGGAGTTCAGACGTGTGC |
| PCR2 Illumina Index 5 | CAAGCAGAAGACGGCATACGAGATCACTGTGTGACTGGAGTTCAGACGTGTGC |
| PCR2 Illumina Index 6 | CAAGCAGAAGACGGCATACGAGATATTGGCGTGACTGGAGTTCAGACGTGTGC |
| PCR2 Illumina Index 7 | CAAGCAGAAGACGGCATACGAGATGATCTGGTGACTGGAGTTCAGACGTGTGC |

Table S2. Candidate loci significantly associated with at least one environmental variable from BayEnv 2.0 and RDA analyses when all 241 fish were included. Median Bayes Factors (BF) are printed for loci identified via BayEnv 2.0. P-values are printed for loci identified via redundancy analysis and regression. In the main text, we randomly removed 9 of 10 fish captured from the same tow on Georges Bank because no other tow displayed a similar level of individual relatedness. Analyses including these individuals identified 22 candidate loci associated with at least one environmental variable. The majority of candidate loci (14 in total) were associated with bottom temperature (64%). Analyses using all 241 fish identified 10 of the 15 candidate loci identified using 232 fish in the main text (67%).

|  |  | | |  | | **Environmental variables** | | | |
| --- | --- | --- | --- | --- | --- | --- | --- | --- | --- |
| Contig Number | | Variant BP | Identified via | | Distance from southern point | | Depth | Bottom Temp | Bottom Salinity |
| 2558 | | 83 | BayEnv2 | | 3.11 | | 3.77 |  | 5.72 |
| 8420 | | 14 | BayEnv2 | | 8.02 | | 7.33 | 6.33 | 3.28 |
| 15075 | | 20 | BayEnv2 | | 4.58 | |  | 5.62 |  |
| 15603 | | 52 | RDA | |  | |  |  | 9.57x10^-4^ |
| 19024 | | 55 | BayEnv2 | |  | | 3.47 |  |  |
| 19728 | | 116 | RDA | |  | | 1.82x10^-4^ | 5.41x10^-5^ |  |
| 30747 | | 44 | BayEnv2 | | 3.53 | | 3.97 | 3.02 | 3.14 |
| 31661 | | 32 | BayEnv2 | | 3.15 | |  | 3.37 |  |
| 37577 | | 42 | BayEnv2 | |  | |  | 3.74 |  |
| 42485 | | 39 | BayEnv2 | | 13.37 | | 9.06 | 7.58 | 10.68 |
| 42614 | | 134 | BayEnv2 | | 6.02 | | 4.14 | 3.47 | 7.13 |
| 43324 | | 33 | BayEnv2 | | 6.71 | |  | 13.33 |  |
| 45459 | | 53 | BayEnv2 | |  | |  | 3.48 |  |
| 50777 | | 49 | BayEnv2 | | 8.91 | | 6.09 | 4.73 | 4.11 |
| 54409 | | 32 | BayEnv2 | |  | |  |  | 7.04 |
| 69416 | | 44 | BayEnv2 | |  | |  | 3.42 |  |
| 70431 | | 68 | BayEnv2 | | 3.41 | | 3.17 |  | 3.60 |
| 77204 | | 133 | BayEnv2 | | 3.26 | | 3.80 |  |  |
| 80216 | | 107 | BayEnv2 | |  | |  | 4.30 |  |
| 83806 | | 70 | BayEnv2 | | 3.15 | | 3.61 |  |  |
| 84811 | | 8 | BayEnv2 | | 3.63 | |  | 3.10 |  |
| 88541 | | 98 | BayEnv2 | |  | | 3.43 |  |  |

Table S3. Sensitivity analysis for parameters estimated using ABC analysis. The first two columns illustrate the trade-off between the ‘# of PCs retained’ and ‘proportion of variance explained’. Parameter estimates for each ABC analysis retaining a different number of principal components (PCs), or summary statistics, are shown by the mode and 95% credible interval. Estimates of the dispersal parameter (DISP) did not differ greatly based on the number of summary statistics used in ABC analysis.

| **# of PCs retained** | **cumulative proportion of variance explained** | **Parameter** | **Mode** | **95% CI** |
| --- | --- | --- | --- | --- |
| 100 | 0.397 | N_e_ north of Cape Hatteras:  POPONE | 726 | 297 - 78235 |
|  |  | N_e_ south of Cape Hatteras:  POPTWO | 745 | 270 - 73418 |
|  |  | Dispersal rate between populations: DISP | 0.30 | 0.031 – 0.49 |
| 450 | 0.630 | N_e_ north of Cape Hatteras:  POPONE | 1628 | 397 - 87237 |
|  |  | N_e_ south of Cape Hatteras:  POPTWO | 3160 | 402 - 89454 |
|  |  | Dispersal rate between populations: DISP | 0.45 | 0.046 – 0.49 |
| 1000 | 0.724 | N_e_ north of Cape Hatteras:  POPONE | 25781 | 608 - 93454 |
|  |  | N_e_ south of Cape Hatteras:  POPTWO | 47413 | 563 - 88145 |
|  |  | Dispersal rate between populations: DISP | 0.41 | 0.056 – 0.49 |
| 8000 | 0.999 | N_e_ north of Cape Hatteras:  POPONE | 52820 | 1451 - 95411 |
|  |  | N_e_ south of Cape Hatteras:  POPTWO | 11545 | 1442 - 92278 |
|  |  | Dispersal rate between populations: DISP | 0.44 | 0.072 – 0.49 |
| 10000 | 1 | N_e_ north of Cape Hatteras:  POPONE | 29888 | 935 - 95126 |
|  |  | N_e_ south of Cape Hatteras:  POPTWO | 40710 | 1056 - 92491 |
|  |  | Dispersal rate between populations: DISP | 0.44 | 0.071 – 0.496 |

Table S4. Candidate loci significantly associated with at least one environmental variable from BayEnv 2.0 and RDA analyses (as indicated by an x), and the corresponding mean regional allele frequency difference between north and south of Cape Hatteras, North Carolina.

| **Contig number** | **Variant BP** | **Regional allele freq difference** | **BayEnv 2.0** | **RDA** |
| --- | --- | --- | --- | --- |
| 2558 | 83 | 0.111 | x |  |
| 8420 | 14 | 0.11 | x | x |
| 15075 | 20 | 0.081 | x |  |
| 19728 | 116 | 0.123 |  | x |
| 27738 | 24 | 0.089 | x |  |
| 31661 | 32 | 0.097 | x |  |
| 35399 | 131 | 0.072 | x | x |
| 37577 | 42 | 0.1 | x |  |
| 38595 | 30 | 0.068 | x |  |
| 42485 | 39 | 0.073 | x |  |
| 42614 | 134 | 0.094 | x |  |
| 54409 | 32 | 0.114 | x | x |
| 61273 | 27 | 0.076 | x |  |
| 69416 | 44 | 0.11 | x |  |
| 75053 | 12 | 0.092 | x |  |

Table S5. Results of BLASTing the contigs containing SNPs associated with at least one environmental variable (Tables 3 & 4) using the nucleotide to nucleotide (megablast) and translated nucleotide to protein BLAST (blastx) algorithms. The *Paralichthys olivaceus* genome and nucleotide collection (nr/nt) databases were searched using the megablast algorithm. The non-redundant protein sequences (nr) database was searched using the blastx algorithm. Only top matches with an e-value ≤1E-4 are reported.

| **Contig number** | **Variant BP** | **Contig sequence** | **Nucleotide to nucleotide BLAST (megablast) against the Japanese flounder genome (taxid: 8255)** | **Nucleotide to nucleotide BLAST (megablast)** | **Translated nucleotide to protein BLAST (blastx)** |
| --- | --- | --- | --- | --- | --- |
| 2558 | 83 | NTGCAGATTATTTCATTTCACGTCCACTAGAGCTTAGAAATAATTTCATCTGAATCCTCAGCGAAGTCTATCAGTGCACTCTAGAATCTGATTAAAGAAATCCCCCATTCTGTCGATATGCGGCCGCCATGCTGCGGGTTTGATACAGATCATTTCATTTCAAATCTCAGCTGGCAAACAGTGGCTCGCAGGAAAN | Flanking DLK1 & BEGAIN genes; NW_017859650.1; e-value = 7E-93 | No match | No match |
| 8420 | 14 | NTGCAGCTGCATTTAGGAGAACACACAGACACCCATGGTTTAAACAAAAGGGAACTAGAGTCACATACAGTAATTTCTGACAAAGCTGGAATATCACATAATATCAAGAAAGCAGACTGGATTTACATAATCCAAACTAATCGAGTTAAATTCAGATGTTTTTTCTCTGTGCTTTGTTTGTTATTCATTGGAATTN | MAP7 gene; NW_017859641.1; e-value = 1E-90 | No match | No match |
| 15075 | 20 | NTGCAGGCAGGCCGGAAGGTTGGTAAGTATAGCAATCTGCTCACTGCCCATCTGGTAGGTGAGAGAGTGCACCCAAATAATAGTGGGATGATTCCCCCTACAACCAGTCATTAGGAGGTTAAATTAGTGCTGAGGCTTAATGGAACTGCACTGGCTGAAGGTAAAGTCTCAGAAATGGCCACCTGTCACACCTAAN | FSTL4 gene; NW_017859651.1; e-value = 1E-90 | No match | No match |
| 19728 | 116 | NTGCAGTTGCATATCTCTGTGGCCACCTGCACACGCTGGGCGGCCTGATGCCTGTGCTCCACAGCCGACACCCTCAAGGCACCCTGGAGCTGGAGCTGGGTGACTGGATGGACAACCGCAGGTATGCAATGCGACATGATCACAAAATACTCATAGCTGTGTGTTTATTACCTCCCTTCCTCTGCTTTTCAGTGAN | TMEM62 gene; NW_017860796.1; e-value = 7E-83 | PREDICTED: *Paralichthys olivaceus* transmembrane protein 62 (tmem62), mRNA; XM_020109200.1; e-value = 1E-52 | PREDICTED: *Paralichthys olivaceus* transmembrane protein 62; XP_019964759.1; e-value = 5E-21 |
| 27738 | 24 | NTGCAGCTTATTTGAACCATTCGGTGCATGAGCAAAAGGCAGATTTACCCAGCTCTGTTAGGATGGTCGGGATCCTAAGGAGAATTTTTTTCTAGATAAGATTACGTAACAAGACTGGAGACTAAGCACTTGCCCAGTAAAGCTTTTGAAGTAAAATTACATTATGGGTTTTGTCCATTGAACCAATATCTGAATN | NBEAL1 gene; NW_017859654.1; e-value = 3E-77 | No match | No match |
| 31661 | 32 | NTGCAGATTACGGACATCATCAAACAGGTAAAGGCGAGGACTTGAATGTAAAAGTAGAATTTATGAATTTGTAGTTTTACAAAAAAAATATTGATTTTTGTGATTCAGAGCTATATGAAGACATTTATGAAGTTGAAACTCAAAAAGGAGAAAATATCAGATGTTCAGAAAGCACTGCGCTCTGGATCTGGAAAAN | PKD2L1 gene; NW_017859642.1; e-value = 3E-91 | PREDICTED: *Paralichthys olivaceus* polycystic kidney disease 2-like 1 protein (LOC109630539), mRNA; XM_020088801.1; e-value = 5E-36 | No match |
| 35399 | 131 | NTGCAGCAACAGTGTGCGCGCGATAAGGGCGCAGCTCTGGAGCGTTGTTTTTTTTTTTTTCTAGATAGCAGTGAAGTTTATGGCACATCAGAGCGCAGTAAAAGCTCCAGCTCCTCTTGAGATATGCAGCCTCTTATCACCGCTGACTGTTGCCTTAAACTTGCCTATACGGACTCCGCTGATCATGTACAGAATN | Flanking SCML4 gene; NW_017859659.1; e-value = 4E-75 | No match | No match |
| 37577 | 42 | NTGCAGAATCCCATGTTAAAAGATGCAAGACTGTATTCTGACTGTATGGGAATAATTAGTGGCTTACCTTCACTATAATGTCAGACATCCTTCTGCTCTGTTTCCTGTGTATCTCACTGGGAGTTCCCAGAATGAAACGTGCCAAAAATGTAATGAAATTTGTCTAATAAAAGGCTTAACCAAGAGTATACCCTCN | ASTN2 gene; NW_017859647.1; e-value = 3E-82 | No match | No match |
| 38595 | 30 | NTGCAGGTTCCCGGGATTGGTGATAAGGGCAGAGGAGGTCAGATAGGTACTGGGGGGCAAGACCATAGAGGGATTTGTAGGTTAGGAGTAGGATTTTGTAATGGATGCAGGACTTGGCAGGAAGCCAGTGAAGTTGGATGAGGGTGGGGGTCATGTGCTGCCAGGGCTTGGTGCAAGTGAGAACCCTGGCAGTTGN | uncharacterized protein LOC109629941; NW_017859654.1; e-value = 3E-67 | No match | PREDICTED: *Notothenia coriiceps* proto-oncogene vav-like; XP_010793755.1; e-value = 2E-19 |
| 42485 | 39 | NTGCAGCAGAAAATTTCACAACATGCTCGTCTTCGCTCCTTTTTTCAGTTTAGTTCTTAGTGTGCTTGTAGTGACGTCGTGCACTCGCTTTTTTCAAATCCAATACCCTGTCTCCTAAGCATGCACAGTGCAGTGAGCCCCCTCCCCCCTCGTGTTTTCTACAGCACAAAAGTGTCCAGTAACAAATACACAAATN | No annotation; NW_017860243.1; e-value = 7E-83 | PREDICTED: *Paralichthys olivaceus* lysine demethylase 7A (kdm7a), mRNA; XM_020107717.1; e-value = 2E-80 | No match |
| 42614 | 134 | NTGCAGTGAGCGGGCAGCAGTCTGGATGCTACATGTCAAAAATGAGTCTGAGATGATTTCAAAAGAGTGGCAGCAGGAGTTAGGGCCAGTTCCTCTACACTGCTCCAGGATCAGGACTGTGCAGCGCCATGACCACGTCAGAGCACATCGTTTTTGGATCCAGGGTATTTAAGTTTGTCACCCAGTGGGATCAGGN | Flanking PDHB & PXK genes; NW_017859667.1; e-value = 3E-91 | PREDICTED: *Paralichthys olivaceus* PX domain containing serine/threonine kinase like (pxk), transcript variant X3, mRNA; XM_020096707.1; e-value = 9E-89 | No match |
| 54409 | 32 | NTGCAGTGGGCCCCGGTTCGAATCCCACATCGGCCGGCCCTTTACTGCGTGTCATTCCCCCCTCTCTCTGCCCCCTGCTTCTTGTCTCTCTTCAACTGTCCTATCATTAAAGGCATAAAAGCCCAAAAAATATACTTACAAAAAAAAAATGCACAAATGTAAAATTTCCATTACATCACATTGAACATGGCAGAGN | Flanking LINGO1 & CSPG4 genes; NW_017859644.1; e-value = 2E-49 | *Larimichthys crocea* genome assembly, chromosome: XVII; LT972183.1;  e-value = 3E-48 | *Larimichthys crocea* E3 ubiquitin-protein ligase HERC2; KKF11948.1;  e-value = 1E-04 |
| 61273 | 27 | NTGCAGTCCTCAGATATGGGTCAACTCACCCTCCTGTACCAGCCTTAGAACAAGCCATTCAGTGTGGCAGCCCCCACCCACAGTTCCTTAAAACACTTTATCCCCAGTTTACATTACCACTATTTTTATTCTGTCCTTGATTTGTTAGTGATTATTGTTATTATCATTATTAAGACAGATTTAAGCCTGTCTTAGN | Flanking SOX5 & BCAT1; NW_017859660.1; e-value = 6E-74 | No match | No match |
| 69416 | 44 | NTGCAGCTGAGCTGTACTCGACCTTTAACATGGAACCTTCCACACTGGAAACATTCCAATGACAGTAACCTCTGCTGTGTTTCCTACAGGTACAAGGAGTTGATGACAGGAAAGACTGCCCGAGAGATCATCGCTATTTTATGGATCCTCTCCTTTATTATCGGCCTCATCCCGTTCTTTGGCTGGAACCTGAAGN | ADORA2B gene; NW_017859651.1; e-value = 7E-73 | PREDICTED: *Paralichthys olivaceus* adenosine A2b receptor (adora2b), transcript variant X2, mRNA; XM_020085548.1;  e-value = 6E-46 | PREDICTED: *Notothenia coriiceps* adenosine receptor A2b; XP_010765545.1;  e-value = 2E-06 |
| 75053 | 12 | NTGCAGACATGAGACCAATGAGCCGGCAGTGATCAGGGCGAGTTAGGAGTTATAACCAGCAGCTGTGCACTTGGTAATGACTCAGACTATAAGTTCAGTCCTGGTCTTCAGTGGAGCGGAGGACGATACCTGGTGCAGCGAGGCCTGACTGGTCTGTTTGTGTTTTTAATCTGAGACAAAGACGGAGAAACCCTGN | Flanking LMCD1 & GMPPB; NW_017859667.1; e-value = 4E-85 | PREDICTED: *Paralichthys olivaceus* LIM and cysteine rich domains 1 (lmcd1), transcript variant X2, mRNA; XM_020096594.1;  e-value = 4E-52 | No match |
